# Supplementary material for: Bioassay Analysis and Molecular Docking Study Revealed the Potential Medicinal Activities of Active Compounds Polygonumins B, C and D from Polygonum minus (Persicaria minor)
Source: Plants (Basel). 2022 Dec 22;12(1):59. doi: 10.3390/plants12010059 (PMC9823858; doi:10.3390/plants12010059)
Supplement: Supplementary file 1 [file plants-12-00059-s001.zip › Figure S4 1H,13C, HMBC, COSY and HSQC of polygonumins D.pdf]

CPM-7
1H of Polygonumins-D in MeOD

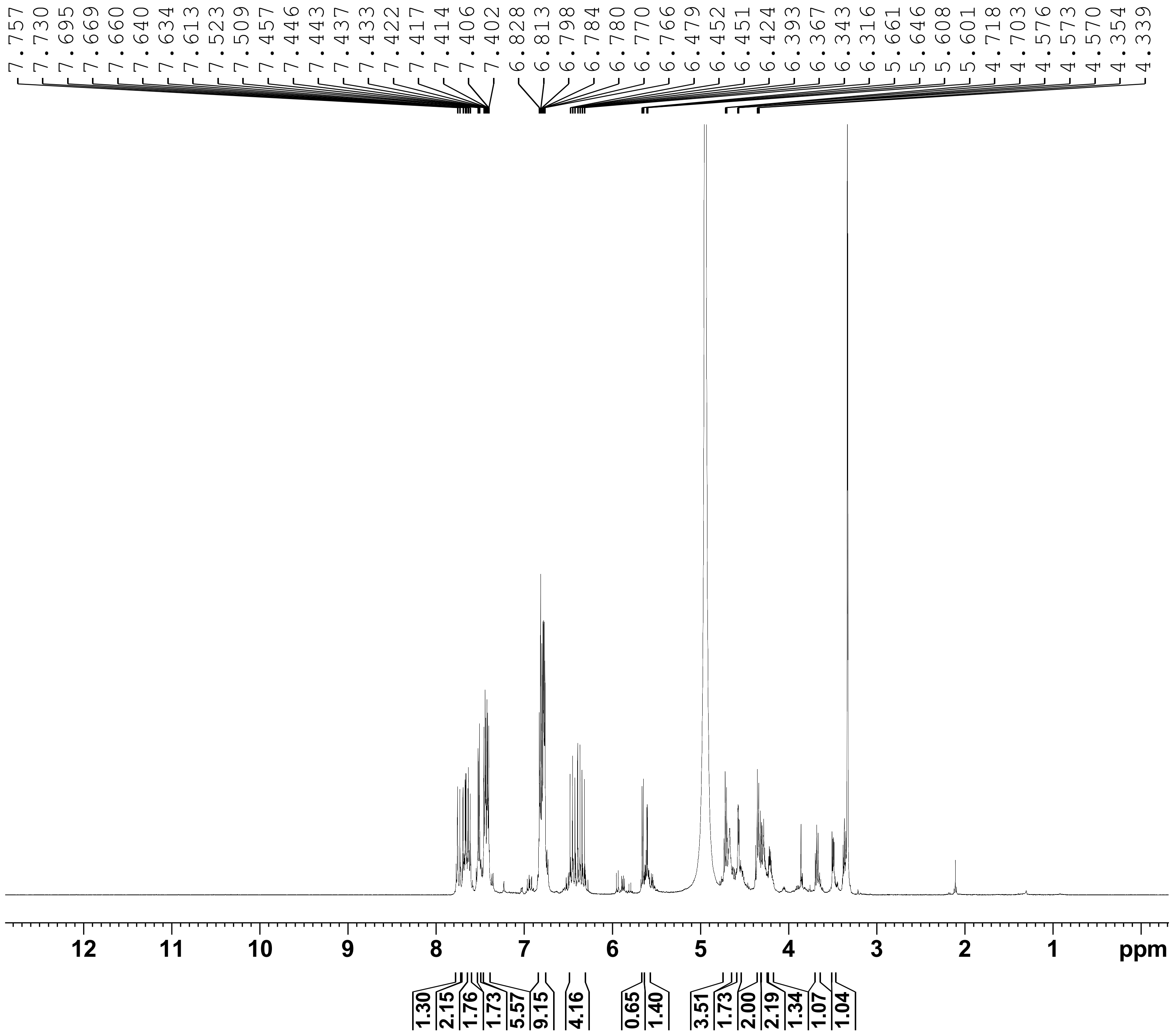

Current Data Parameters
NAME Andy_N-Y-a2
EXPNO 1
PROCNO 1

F2 - Acquisition Parameters
Date_ 20130718
Time 21.54
INSTRUM spect
PROBHD 5 mm CPTCI 1H-
PULPROG zg30
TD 65536
SOLVENT MeOD
NS 16
DS 2
SWH 12335.526 Hz
FIDRES 0.188225 Hz
AQ 2.6563926 sec
RG 32
DW 40.533 usec
DE 6.50 usec
TE 297.0 K
D1 1.00000000 sec
TD0 1

======== CHANNEL f1 ========
NUC1 1H
P1 10.00 usec
PL1 4.00 dB
PL1W 5.26999998 W
SFO1 600.3037071 MHz

F2 - Processing parameters
SI 32768
SF 600.3000013 MHz
WDW EM
SSB 0
LB 0.30 Hz
GB 0
PC 1.00

CPM-7
13C of Polygonumins-D in MEOD

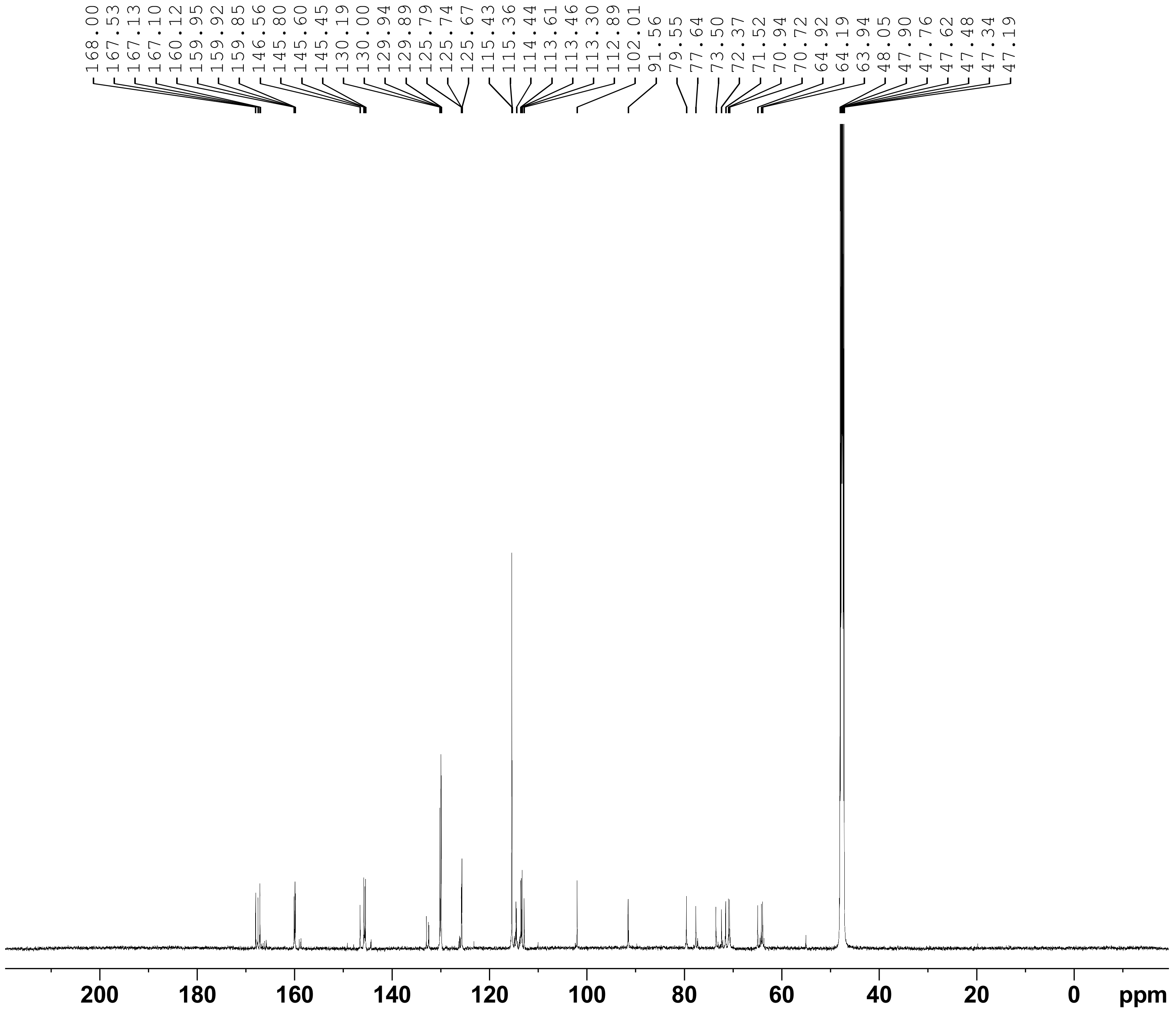

Current Data Parameters
NAME Andy_N-Y-a2
EXPNO 20
PROCNO 1

F2 - Acquisition Parameters
Date_ 20130718
Time 22.10
INSTRUM spect
PROBHD 5 mm CPTCI 1H-
PULPROG zgpg30
TD 65340
SOLVENT MeOD
NS 20480
DS 4
SWH 36057.691 Hz
FIDRES 0.551847 Hz
AQ 0.9060480 sec
RG 203
DW 13.867 usec
DE 50.34 usec
TE 296.9 K
D1 2.00000000 sec
D11 0.03000000 sec
TD0 1

======== CHANNEL f1 ========
NUC1 13C
P1 13.00 usec
PL1 0.20 dB
PL1W 84.43891907 W
SFO1 150.9606491 MHz

======== CHANNEL f2 ========
CPDPRG[2 waltz16
NUC2 1H
PCPD2 80.00 usec
PL2 4.00 dB
PL12 21.73 dB
PL13 14.00 dB
PL2W 5.26999998 W
PL12W 0.08888135 W
PL13W 0.52700001 W
SFO2 600.3024012 MHz

F2 - Processing parameters
SI 32768
SF 150.9455550 MHz
WDW EM
SSB 0
LB 2.00 Hz
GB 0
PC 1.40

CPM-7
HMBC of Polygonimuns-D in MeOD

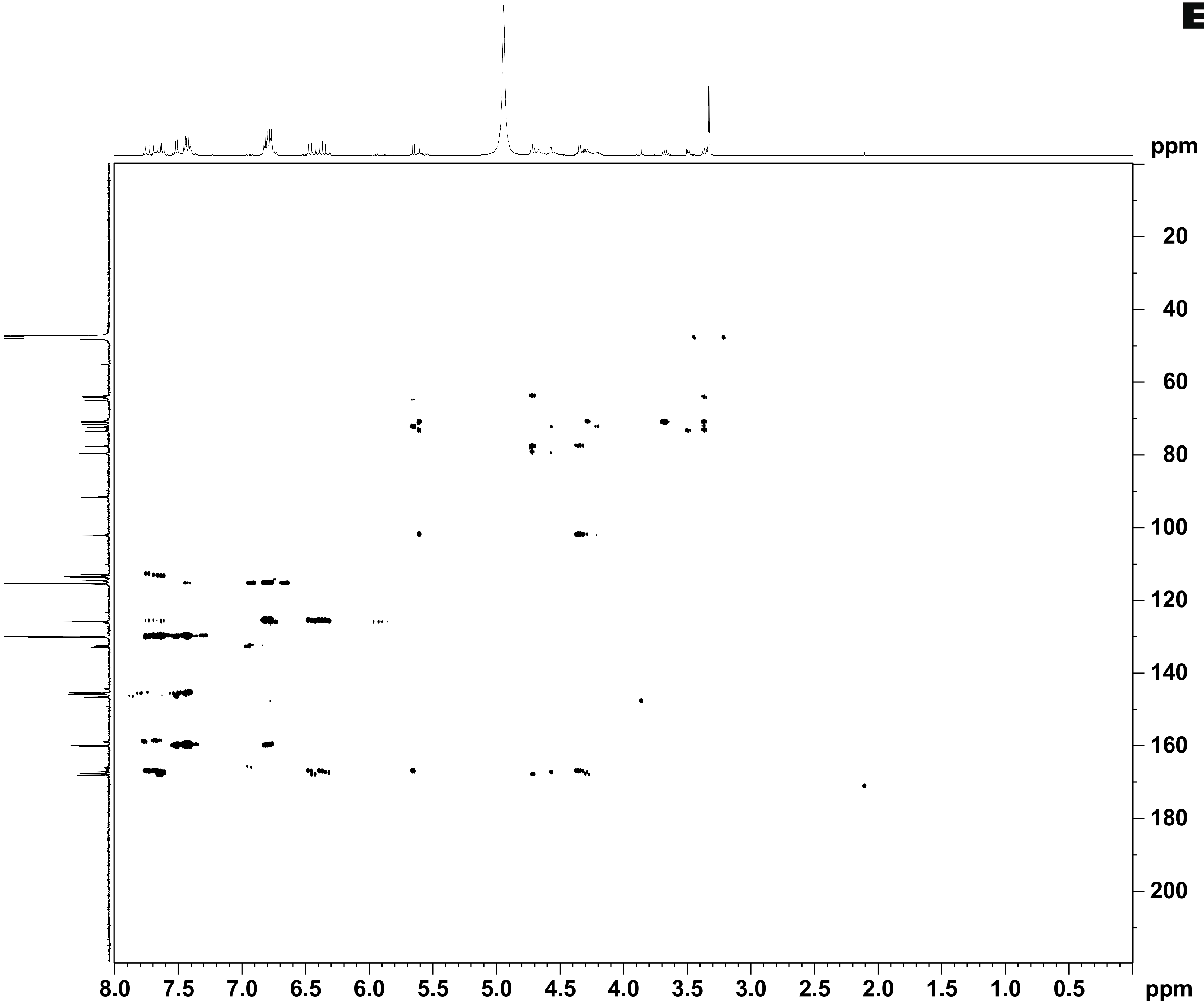


```
Current Data Parameters
NAME          Andy_N-Y-a2
EXPNO                   5
PROCNO                  1

F2 - Acquisition Parameters
Date_            20130719
Time                22.50
INSTRUM             spect
PROBHD    5 mm CPTCI 1H-
PULPROG     hmbcgplpndqf
TD                   4096
SOLVENT              MeOD
NS                     64
DS                     16
SWH              4807.692 Hz
FIDRES           1.173753 Hz
AQ              0.4259840 sec
RG                    203
DW                104.000 usec
DE                   6.50 usec
TE                  296.8 K
CNST2         145.0000000
CNST13         10.0000000
D0             0.00000300 sec
D1             1.50000000 sec
D2             0.00344828 sec
D6             0.05000000 sec
D16            0.00020000 sec
IN0            0.00001505 sec

======== CHANNEL f1 ========
NUC1                   1H
P1                  10.00 usec
P2                  20.00 usec
PL1                  4.00 dB
PL1W           5.26999998 W
SFO1          600.3024012 MHz

======== CHANNEL f2 ========
NUC2                  13C
P3                  12.00 usec
PL2                  0.20 dB
PL2W          84.43891907 W
SFO2          150.9621590 MHz

====== GRADIENT CHANNEL =====
GPNAM[1]         SINE.100
GPNAM[2]         SINE.100
GPNAM[3]         SINE.100
GPZ1                50.00 %
GPZ2                30.00 %
GPZ3                40.10 %
P16               1000.00 usec

F1 - Acquisition parameters
TD                    256
SFO1             150.9622 MHz
FIDRES         259.466217 Hz
SW                220.000 ppm
FnMODE                 QF

F2 - Processing parameters
SI                   2048
SF            600.2999988 MHz
WDW                  SINE
SSB                     0
LB                      0 Hz
GB                      0
PC                   1.40

F1 - Processing parameters
SI                   1024
MC2                    QF
SF            150.9455900 MHz
WDW                  SINE
SSB                     0
LB                      0 Hz
GB                      0
```

CPM-7
Cosy of Polygonumins-D in MeOD

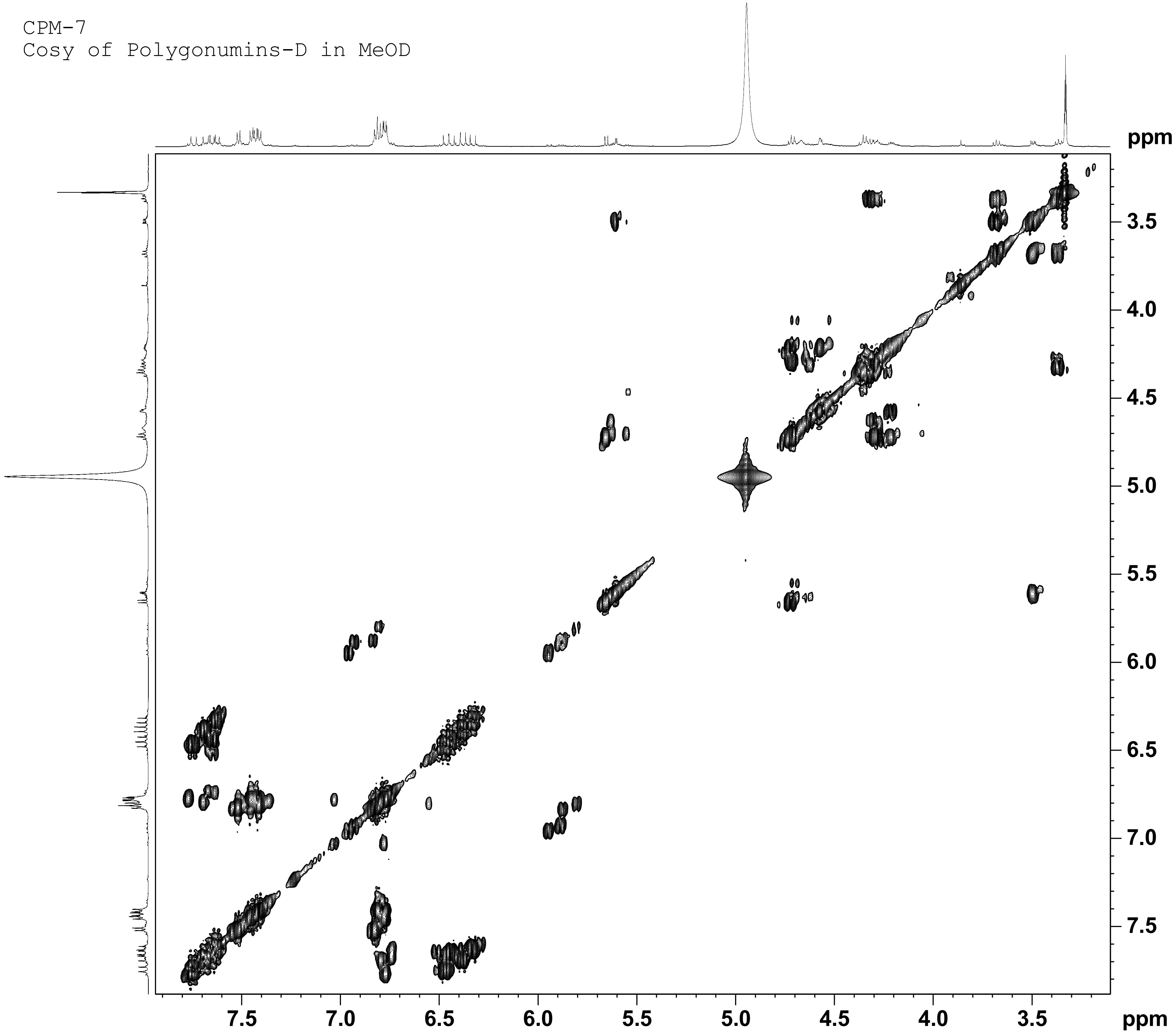

Current Data Parameters

NAME	Andy_N-Y-a2
EXPNO	2
PROCNO	1

F2 - Acquisition Parameters

Date_	20130720
Time	11.37
INSTRUM	spect
PROBHD	5 mm CPTCI 1H-
PULPROG	cosygpqf
TD	2048
SOLVENT	MeOD
NS	32
DS	16
SWH	4807.692 Hz
FIDRES	2.347506 Hz
AQ	0.2129920 sec
RG	203
DW	104.000 usec
DE	6.50 usec
TE	296.9 K
D0	0.00000300 sec
D1	2.00000000 sec
D13	0.00000400 sec
D16	0.00020000 sec
IN0	0.00020825 sec

======== CHANNEL f1 ========

NUC1	1H
P0	10.00 usec
P1	10.00 usec
PL1	4.00 dB
PL1W	5.26999998 W
SFO1	600.3024012 MHz

====== GRADIENT CHANNEL =====

GPNAM[1]	SINE.100
GPZ1	80.00 %
P16	1000.00 usec

F1 - Acquisition parameters

TD	256
SFO1	600.3024 MHz
FIDRES	37.518902 Hz
SW	8.000 ppm
FnMODE	QF

F2 - Processing parameters

SI	1024
SF	600.2999984 MHz
WDW	SINE
SSB	0
LB	0 Hz
GB	0
PC	1.40

F1 - Processing parameters

SI	1024
MC2	QF
SF	600.2999998 MHz
WDW	SINE
SSB	0
LB	0 Hz
GB	0

CPM-7
HSQC of Polygonumins-D in MeOD

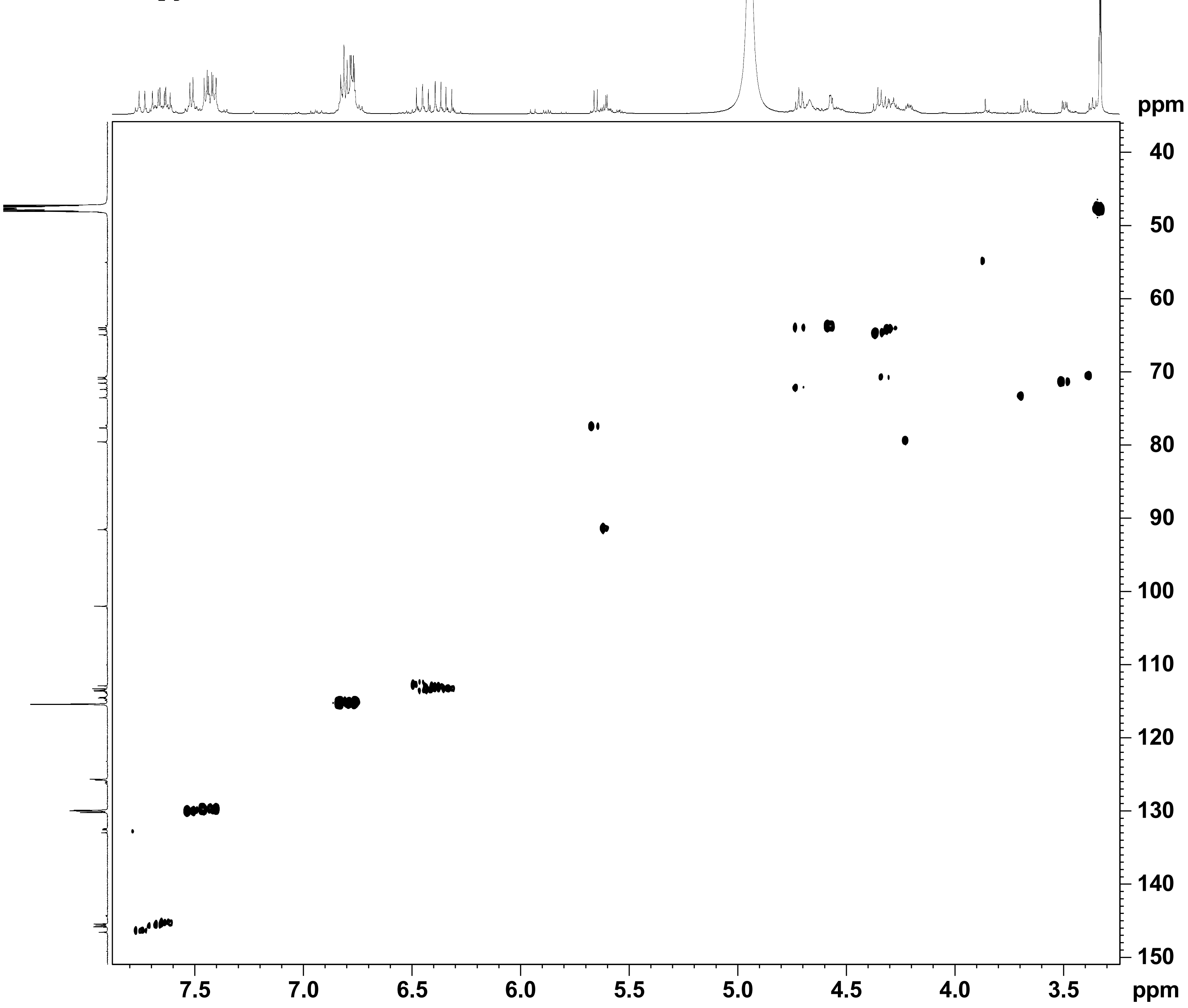

Current Data Parameters

NAME	Andy_N-Y-a2
EXPNO	4
PROCNO	1

F2 - Acquisition Parameters

Date_	20130720
Time	7.53
INSTRUM	spect
PROBHD	5 mm CPTCI 1H-
PULPROG	hsqcedetgp
TD	1024
SOLVENT	MeOD
NS	32
DS	16
SWH	4807.692 Hz
FIDRES	4.695012 Hz
AQ	0.1064960 sec
RG	203
DW	104.000 usec
DE	6.50 usec
TE	297.0 K
CNST2	145.0000000
D0	0.00000300 sec
D1	1.50000000 sec
D4	0.00172414 sec
D11	0.03000000 sec
D13	0.00000400 sec
D16	0.00020000 sec
D21	0.00345000 sec
IN0	0.00001950 sec
ZGOPTNS	

======== CHANNEL f1 ========

NUC1	1H
P1	10.00 usec
P2	20.00 usec
P28	0 usec
PL1	4.00 dB
PL1W	5.26999998 W
SFO1	600.3024012 MHz

======== CHANNEL f2 ========

CPDPRG[2	garp
NUC2	13C
P3	12.00 usec
P4	24.00 usec
PCPD2	55.00 usec
PL2	0.20 dB
PL12	12.42 dB
PL2W	84.43891907 W
PL12W	5.06457090 W
SFO2	150.9568759 MHz

====== GRADIENT CHANNEL =====

GPNAM[1]	SINE.100
GPNAM[2]	SINE.100
GPZ1	80.00 %
GPZ2	20.10 %
P16	1000.00 usec

F1 - Acquisition parameters

TD	256
SFO1	150.9569 MHz
FIDRES	200.489594 Hz
SW	170.000 ppm
FnMODE	Echo-Antiecho

F2 - Processing parameters

SI	1024
SF	600.2999962 MHz
WDW	SINE
SSB	0
LB	0 Hz
GB	0
PC	1.40

F1 - Processing parameters

SI	1024
MC2	echo-antiecho
SF	150.9455924 MHz
WDW	SINE
SSB	0
LB	0 Hz
GB	0
