# Supplementary material for: Bioassay Analysis and Molecular Docking Study Revealed the Potential Medicinal Activities of Active Compounds Polygonumins B, C and D from Polygonum minus (Persicaria minor)
Source: Plants (Basel). 2022 Dec 22;12(1):59. doi: 10.3390/plants12010059 (PMC9823858; doi:10.3390/plants12010059)
Supplement: Supplementary file 1 [file plants-12-00059-s001.zip › Figure S5 Hydrophobicity surfaceview of polygonumins derivatives with proteins.pdf]

a)

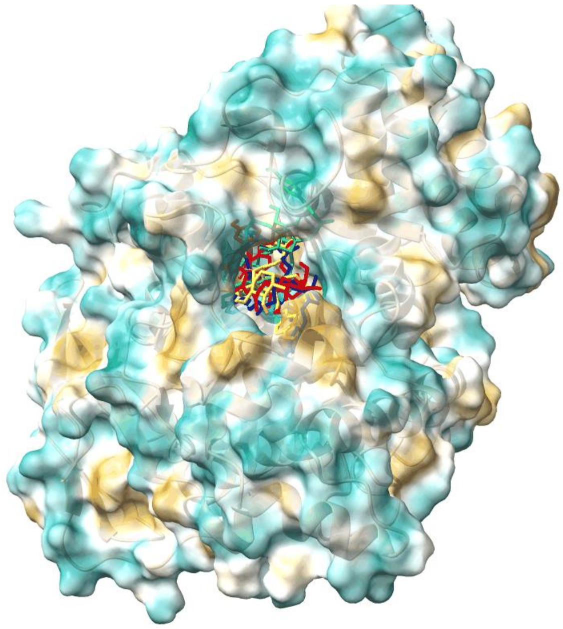

b)

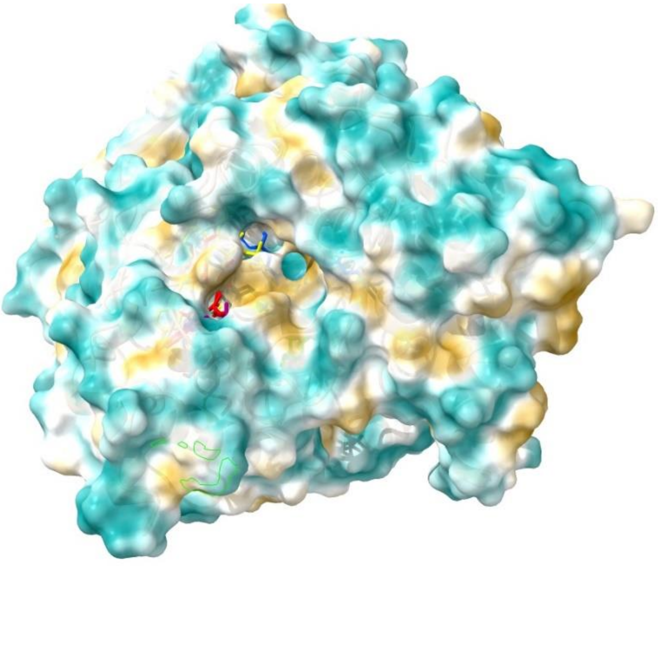

Figure S5. Hydrophobicity surface view of polygonumins derivatives with a) BChE protein b) AChE protein (polygonumins A: red stick, polygonumins B: blue stick, polygonumins C: yellow stick and polygonumins D: green stick, tacrine: magenta). Analysis of hydrophobicity was performed by ChimeraX (gold: hydrophobic, white: intermediate, cyan: hydrophilic).
