# Supplementary material for: Bioassay Analysis and Molecular Docking Study Revealed the Potential Medicinal Activities of Active Compounds Polygonumins B, C and D from Polygonum minus (Persicaria minor)
Source: Plants (Basel). 2022 Dec 22;12(1):59. doi: 10.3390/plants12010059 (PMC9823858; doi:10.3390/plants12010059)
Supplement: Supplementary file 1 [file plants-12-00059-s001.zip › Figure S7 Two dimensional illustration showing the interaction of AChE.pdf]

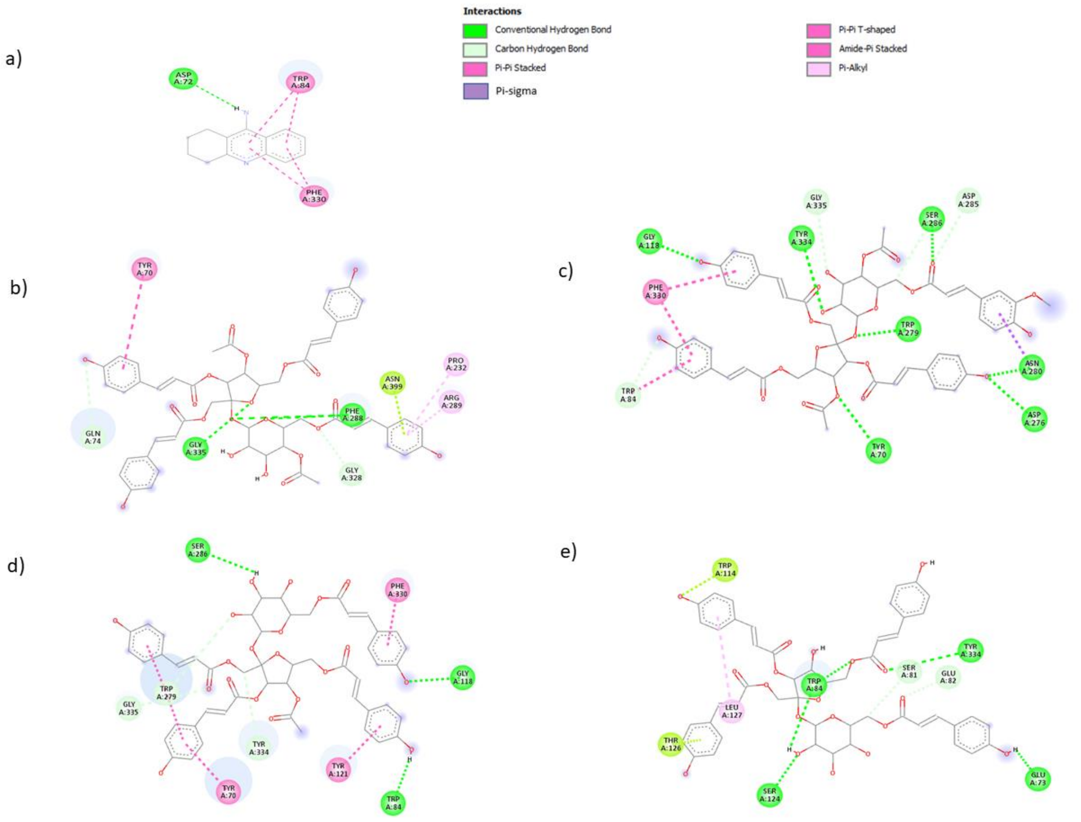

Figure S7. Two-dimensional illustrations showing the interaction of AChE protein with a) tacrine b) polygonumins A, c) polygonumins B, d) polygonumins C and e) polygonumins D. Interaction analysis was done using BIOVIA discovery studio. The colors of the dotted lines explain the types of interactions: hydrophobic (pink), hydrogen bond (green) and π-sigma (purple).
