# Supplementary material for: Bioassay Analysis and Molecular Docking Study Revealed the Potential Medicinal Activities of Active Compounds Polygonumins B, C and D from Polygonum minus (Persicaria minor)
Source: Plants (Basel). 2022 Dec 22;12(1):59. doi: 10.3390/plants12010059 (PMC9823858; doi:10.3390/plants12010059)
Supplement: Supplementary file 1 [file plants-12-00059-s001.zip › Table S1 NMR data of Polygonumins Derivatives .pdf]

Table S1. Comparison of NMR data for polygonumins A, B, C, D, vanicoside A and F

No of C	Polygonumins A* (**CPM-3**)		Vanicoside F**	Vanicoside A***		Polygonumins B (**CPM-1**)		Polygonumins C (**CPM-5**)		Polygonumins D (**CPM-7**)	
	^{13}C (75 MHz)	^{1}H (600 MHz)	^{13}C (100 MHz)	^{13}C (75 MHz)	^{1}H (300 MHz)	^{13}C (75 MHz)	^{1}H (600 MHz)	^{13}C (75 MHz)	^{1}H (600 MHz)	^{13}C (75 MHz)	^{1}H (600 MHz)
fructose											
1	65.0	4.21 (*m*, 1H)<br>4.41 (*m*, 1H)	65.4	62.80	4.25, 4.6 m	65.0	4.38 (*d*, 4.4, 1H)<br>4.20 (*d*, 12.0, 1H)	64.7	4.2 (d, 11.6, 1H)<br>4.33 (d, 11.6, 1H)	64.9	4.33 (*d*, 9.24, 2H)
2	102.7	-	103.5	102.12	-	102.6	-	102.4	-	102.0	-
3	78.2	5.62 (*d*, 7.8, 1H)	81.0	77.64	5.61 d (8.4)	78.2	5.62 (*d*, 7.9, 1H)	78.0	5.63 (d, 8.3, 1H)	77.6	5.68 (*d*, 5.96, 1H)
4	72.7	4.83 (*ddd*, 13.2, 10.2, 3.6, 1H)	74.3	72.65	4.65 m	72.6	4.82 (dd, 3.7, 10.8, 1H)	73.0	4.68 (t, 11.6, 1H)	72.4	4.77 (*t*, 5.96, 1H)
5	80.3	4.31 (*m*, 1H)	81.9	79.82	4.3 m	80.3	4.30 (t, 8.0, 1H)	80.1	4.30 (q, 6.8, 11.0, 1H)	80.0	4.30 (*q*, 12.0, 3.78, 1H)
6	64.2	4.61 (*dd*, 12.0, 3.0, 1H),<br>4.47 (*m*, 1H)	72.1	64.55	4.6 m	64.3	4.48 (d, 6.8 1H)<br>4.62 (dd, 3.4, 12.1, 1H)	64.5	4.55 (d, 15.4, 1H)<br>4.61 (d, 12.1, 1H)	64.2	4.59 (*d*, 7.0, 1H)<br>4.27 (*d*, 6.5, 1H)
glucose											
1'	89.3	5.77 (*d*, 3.6, 1H)	90.6	89.18	5.66 d (3.6)	89.2	5.76 (d, 3.7, 1H)	89.5	5.70 (d, 3.5, 1H)	91.6	5.59 (*d*, 3.64, 1H)
2'	73.2	4.67 (*t*, 7.8, 1H)	74.2	73.01	4.69 m	73.3	4.68 (t, 8.0, 1H)	73.2	4.73 (t, 4.6, 1H)	73.5	3.74 (*t*, 11.41, 1H)
3'	68.6	4.47 (*m*, 1H)	72.2	70.79	3.9 m	68.6	4.50 (t, 7.1, 1H)	71.0	3.96 (t, 4.7, 1H)	71.0	3.41 (*t*, 9.45, 1H)
4'	71.4	4.98 (*t*, 10.2, 1H)	79.4	70.66	3.51 dd (9.4, 9.4)	71.4	4.96 (t, 9.4, 1H)	71.0	3.52 (br-t, 5.1, 1H)	71.5	3.50 (*br-t*, 8.75, 1H)
5'	68.8	4.11 (*br-t*, 9.6, 1H)	72.4	70.79	4.3 m	68.8	4.10 (q,18.0, 9.6, 1H)	72.9	4.34 (m, 1H)	70.7	4.36 (*q*, 14.2, 9.03, 1H)
6'	63.1	4.37 (*m*, 1H)<br>4.18 (*m*, 1H)	65.5	64.49	4.3 m<br>4.35 m	63.1	4.21 (d, 4.9, 1H)<br>4.41 (dd, 2.2, 12.2, 1H)	64.0	4.3 (d, 11.1, 1H)<br>4.59 (d, 15.4, 1H)	63.9	4.72 (*d*, 10.2, 2H)
feruloyl moiety											
1''	126.1	-	127.7	125.69	-	126.5	-	126.2	-	125.8	-
2''	130.21	7.55 (*d*, 8.4, 1 H)	111.5	110.08	7.33 d (1.8)	110.4	7.38 (s, 1H)	130.21	7.55 (d, 8.8 1H)	130.21	7.56-753 (*m*, 2H)
3''	115.9	6.94 (*d*, 8.4, 1 H)	150.6	148.97b		149.3	-	115.8	6.93-6.87 (m, 1H)	115.81	6.91-6.86 (*m*, 2H)
4''	159.81	-	149.3	147.62		147.9	-	159.8	-	159.85	-
5''	115.9	6.94 (*d*, 8.4, 1 H)	114.2	114.94	6.81	116.0	6.90 (d, 8.4, 1H)	115.8	6.93-6.87 (m, 1H)	115.42	6.91-6.86 (*m*, 2H)
6''	130.21	7.55 (*d*, 8.4, 1 H)	124.5	123.24	7.11 dd (1.8, 8.2)	123.4	7.15 (d, 8.2, 1H)	130.21	7.56 (d, 8.8 1H)	129.88	7.56-753 (*m*, 2H)
7''	146.1	7.78 (*d*, 15.6, 1 H)	148.0	-	7.58 d (16)	146.1	7.78 (d, 15.8, 1H)	146.0	7.76 (d, 16.0, 1H)	146.6	7.76 (*d*, 8.0, 1H)
8''	113.6	6.51 (*d*, 14.4, 1 H)	116.3	113.41	6.33 d (16)	115.1	6.54 (d, 15.9, 1H)	113.7	6.52 (d, 16.0, 1H)	112.88	6.43 (*d*, 8.0, 1H)
9''	166.0	-	169.2	165.93	-	166.0	-	166.0	-	167.1	-
3''-OCH_3	-	-	56.4	55.25	3.85 s	55.5	3.91 (s, 3H)	-	-		
p-coumaroyl moieties											
1''' - 1'''''	126.04	-	127.7	125.77	-	126.0	-	126.1	-	125.7	-
	126.0	-	127.1	125.84	-	125.96	-	126.0	-	125.66	-
	125.9	-	127.0	126.38	-	125.9	-	125.9	-	125.66	-
2/6''' – 2/6'''''	130.24	7.57 (*d*, 8.4, 2 H)	131.6	130.02	7.45–7.65 m	130.2	7.54 (d, 8.64, 2H)	130.2	7.62 (d, 8.3, 2H)	129.9	7.56-753 (*m*, 2H)
	130.3	7.57 (*d*, 8.4, 2 H)	131.3	130.06		130.23	7.56 (d, 8.64, 2H)	130.21	7.58 (d,7.86 2H)	130.0	7.56-753 (*m*, 2H)
	130.4	7.55 (*d*, 8.4, 2 H)	131.2	130.31		130.4	7.56 (d, 8.58, 2H)	130.5	7.56 (d, 8.8 2H)	130.2	7.60 (*d*, 8.47, 2H)
3/5''' – 3/5'''''	115.8	6.90 (*m*, 2 H)	116.8	115.63	6.85–6.89 m	115.84	6.89 (d, 8.4, 2H)	115.8	6.93-6.87 (m, 2H)	115.42	6.91-6.86 (*m*, 2H)
	115.9	6.90 (*m*, 2 H)	116.8	115.64		115.84	6.89 (d, 8.4, 2H)	115.84	6.93-6.87 (m, 2H)	115.42	6.91-6.86 (*m*, 2H)
	116.0	6.94 (*d*, 8.4, 2 H)	116.8	115.73		116.0	6.94 (d, 8.52, 2H)	116.0	6.93-6.87 (m, 2H)	115.36	6.91-6.86 (*m*, 2H)
4''' – 4'''''	159.84	-	161.5	159.53	-	159.85	-	159.74	-	159.92	-
	159.9	-	162.3	159.63	-	159.92	-	159.8	-	160.0	-
	160.2	-	161.2	159.81	-	160.2	-	159.9	-	160.1	-
7''' – 7'''''	145.3	7.72 (*d*, 15.6, 1 H)	148.0	144.92	7.62 d (16)	145.5	7.71 (d,15.96, 2H)	145.2	7.73 (d, 16.3, 1H)	145.8	7.69 (*d*, 8.0, 1H)
	145.2	7.68 (*d*, 10.2, 1 H)	147.2	145.91	7.64 d (16)	145.3	7.67 (d, 9.18, 2H)	145.0	7.67 (d, 16.6, 1H)	145.6	7.67 (*d*, 8.0, 1H)
	145.1	7.64 (*dd*, 13.2, 1.2, 1 H)	146.9	145.10	7.72 d (16)	145.1	7.65 (dd, 1.74, 8.28, 2H)	144.9	7.65 (d, 12.8, 1H)	144.4	7.73 (*d*, 8.0, 1H)
8''' – 8'''''	114.1	6.48 (*d*, 13.8, 1 H)	116.3	113.87	6.40 d (16)	114.1	6.51 (d, 15.9, 1H)	114.2	6.49 (d, 16.0, 1H)	114.29	6.45 (*d*, 8.0, 1H)
	114.2	6.43 (*d*, 12.6, 1 H)	115.2	113.97	6.45 d (16)	114.2	6.45 (d, 18.0, 1H)	114.3	6.46 (d, 16.1, 1H)	113.46	6.52 (*d*, 8.0, 1H)
	114.3	6.42 (*d*, 16.2, 1 H)	114.8	114.53	6.53 d (16)	114.5	6.40 (d, 15.96, 1H)	114.6	6.40 (d, 16.0, 1H)	113.61	6.61 (*d*, 8.0, 1H)
9''' – 9'''''	166.04	-	169.2	165.96	-	166.03	-	166.1	-	167.13	-
	166.5	-	168.9	166.46	-	166.5	-	166.5	-	167.5	-
	166.6	-	168.3	166.64	-	166.6	-	166.8	-	168.0	-
acetyl moieties											
1''''''	170.1	-	-	170.12 (2')	-	169.6	-	-	-	-	-
2''''''	20.0	1.99 (s, 3 H)	-	20.03 (1)	2.06 s	20.1	2.0 (s, 3H)	-	-	-	-
1'''''''	169.6	-	172.7	-	-	170.1	-	170.2	-	-	-
2'''''''	20.1	1.99 (s, 3 H)	21.05	-	-	20.0	2.1 (s, 3H)	20.15	2.1 (s, 3H)	-	-

Chemical structure of polygonumins derivatives from *Polygonum minus Huds*, vanicosides F and vanicosides A.

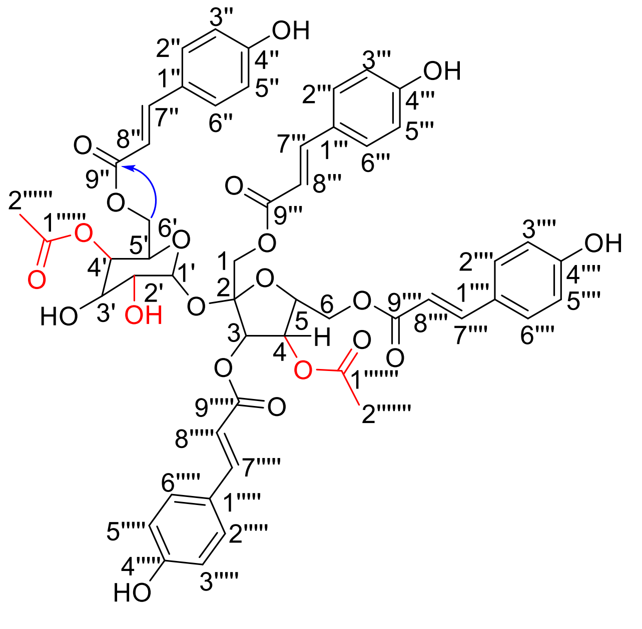	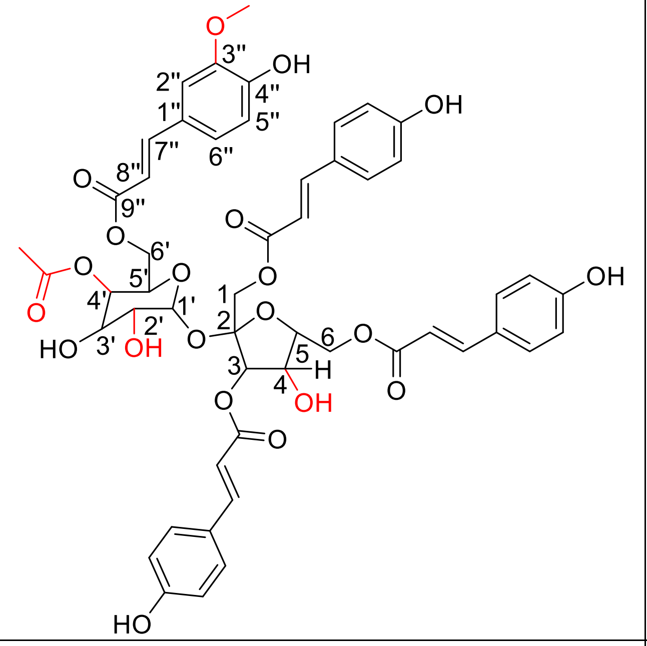	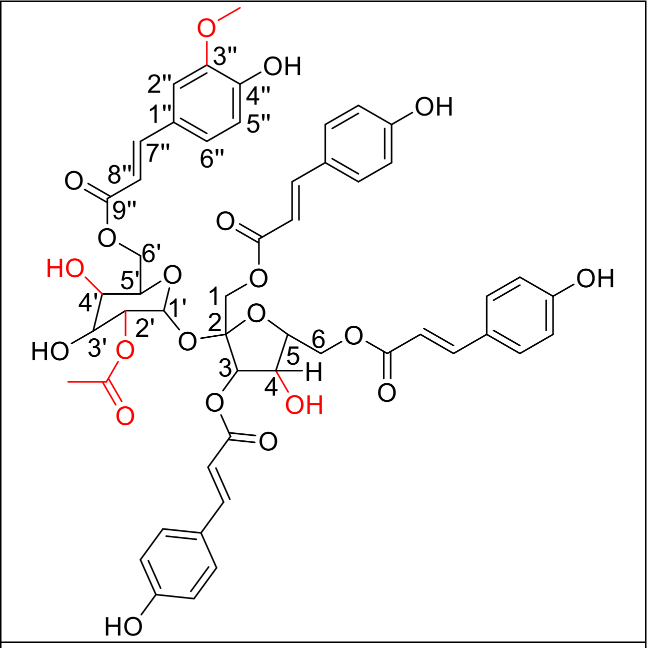
**Polygonumins A** (**CPM-3**) $[M]^+$ m/z 1010, $C_{52}H_{50}O_{21}$	Vanicoside F**	Vanicoside A*
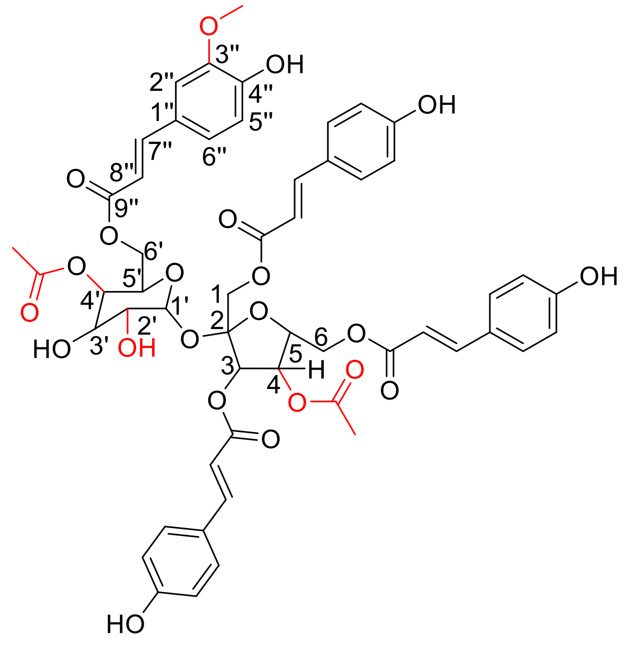	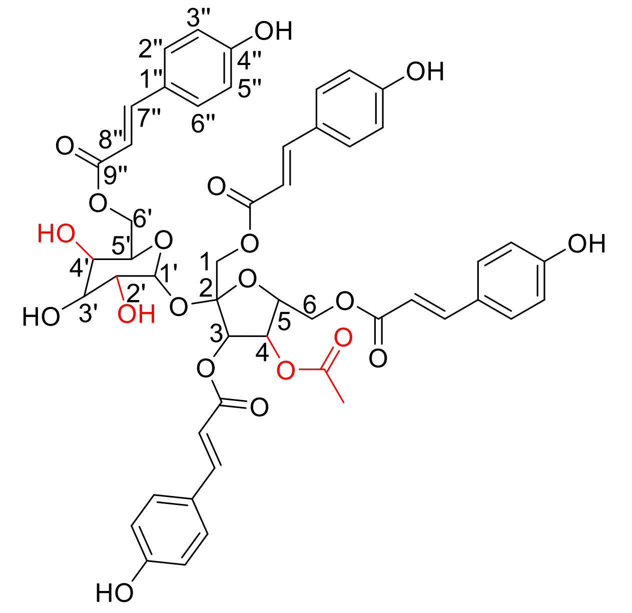	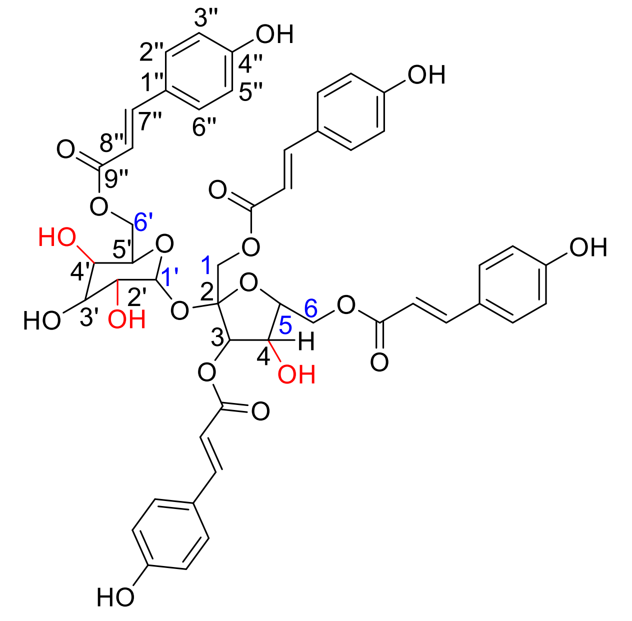
**Polygonumins B** (**CPM-1**) $[M]^+$ m/z 1040, $C_{53}H_{52}O_{22}$	**Polygonumins C** (**CPM-5**) $[M]^+$ m/z 968, $C_{50}H_{48}O_{21}$	**Polygonumins D** (**CPM-6**) $[M]^+$ m/z 944, $C_{48}H_{48}O_{20}$
