# Supplementary material for: Bioassay Analysis and Molecular Docking Study Revealed the Potential Medicinal Activities of Active Compounds Polygonumins B, C and D from Polygonum minus (Persicaria minor)
Source: Plants (Basel). 2022 Dec 22;12(1):59. doi: 10.3390/plants12010059 (PMC9823858; doi:10.3390/plants12010059)
Supplement: Supplementary file 1 [file plants-12-00059-s001.zip › Figure S1 1H, 13C, HMBC, COSY and HSQC of polygonumins A.pdf]

CPM-3
1H of Polygonumins-A in Acetone

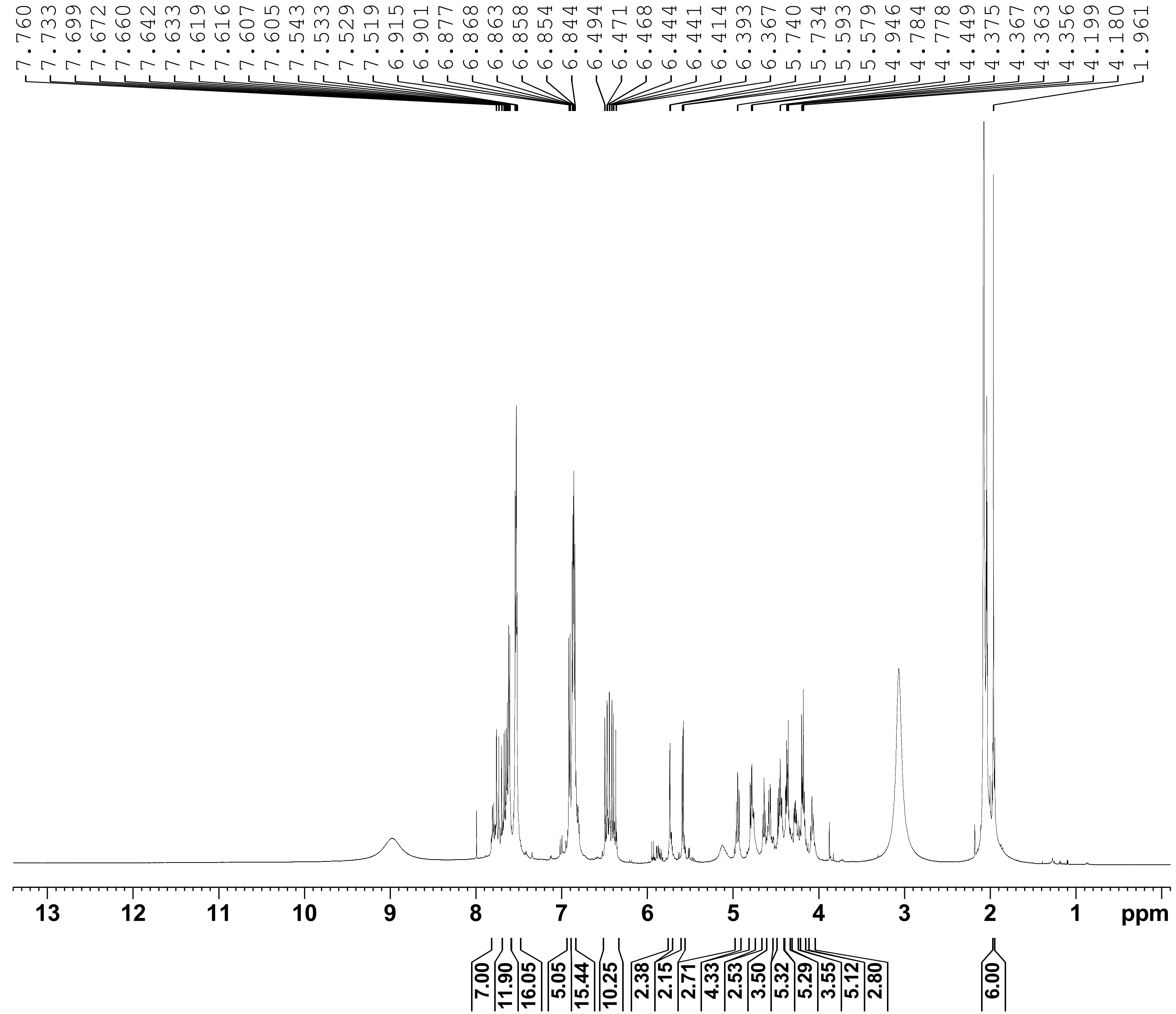

Current Data Parameters

NAME	CPM-3
EXPNO	1
PROCNO	1

F2 - Acquisition Parameters

Date_	20100824
Time	16.50
INSTRUM	spect
PROBHD	5 mm CPTCI 1H-
PULPROG	zg30
TD	65536
SOLVENT	Acetone
NS	16
DS	2
SWH	12335.526 Hz
FIDRES	0.188225 Hz
AQ	2.6563926 sec
RG	20.2
DW	40.533 usec
DE	6.50 usec
TE	303.2 K
D1	1.00000000 sec
TD0	1

======== CHANNEL f1 ========

NUC1	1H
P1	8.00 usec
PL1	4.00 dB
PL1W	5.26999998 W
SFO1	600.1337060 MHz

F2 - Processing parameters

SI	32768
SF	600.1300188 MHz
WDW	EM
SSB	0
LB	0.30 Hz
GB	0
PC	1.00

CPM-3
13C of Polygonumins-A in Acetone

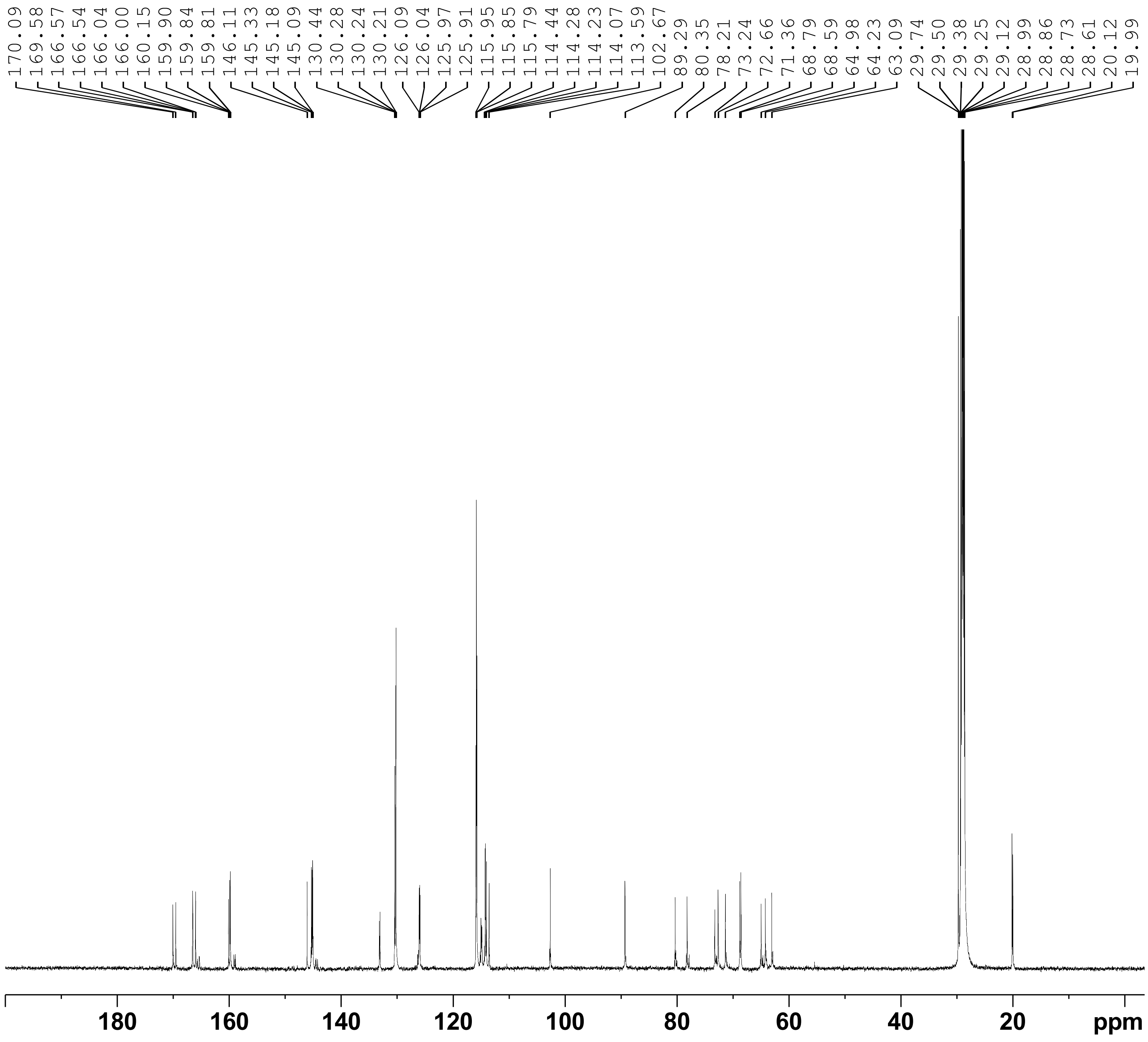

Current Data Parameters
NAME CPM-3
EXPNO 20
PROCNO 1

F2 - Acquisition Parameters
Date_ 20100902
Time 15.12
INSTRUM spect
PROBHD 5 mm CPTCI 1H-
PULPROG zgpg30
TD 65340
SOLVENT Acetone
NS 879
DS 4
SWH 36057.691 Hz
FIDRES 0.551847 Hz
AQ 0.9060480 sec
RG 203
DW 13.867 usec
DE 50.55 usec
TE 303.2 K
D1 2.00000000 sec
D11 0.03000000 sec
TD0 1

======== CHANNEL f1 ========
NUC1 13C
P1 12.00 usec
PL1 0.20 dB
PL1W 84.43891907 W
SFO1 150.9178988 MHz

======== CHANNEL f2 ========
CPDPRG[2 waltz16
NUC2 1H
PCPD2 80.00 usec
PL2 4.00 dB
PL12 24.00 dB
PL13 28.00 dB
PL2W 5.26999998 W
PL12W 0.05270000 W
PL13W 0.02098025 W
SFO2 600.1324005 MHz

F2 - Processing parameters
SI 32768
SF 150.9028090 MHz
WDW EM
SSB 0
LB 2.00 Hz
GB 0
PC 1.40

CPM-3
Polygonumins A, C-H HMBC

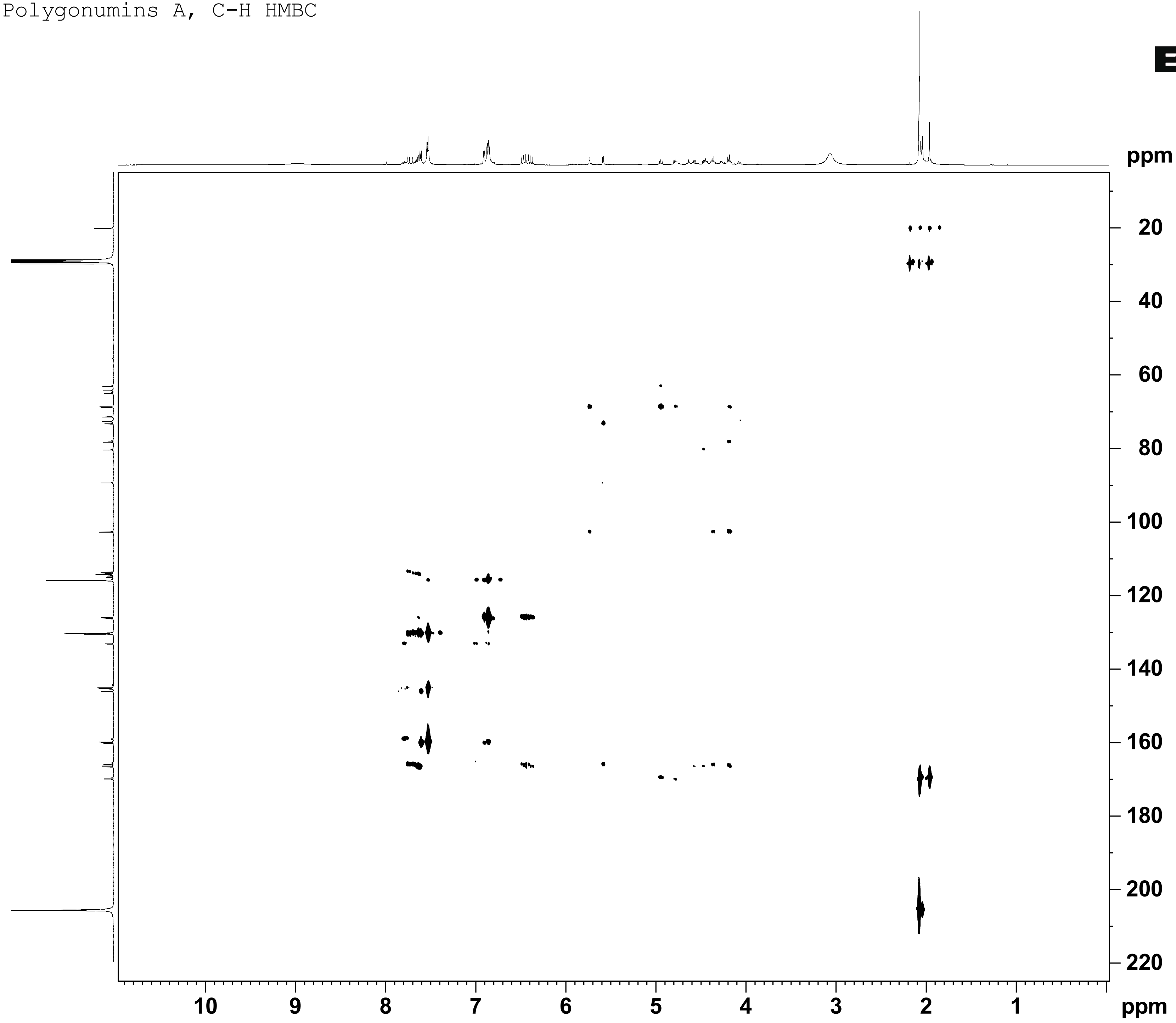

Current Data Parameters
NAME CPM-3
EXPNO 5
PROCNO 1

F2 - Acquisition Parameters
Date_ 20101008
Time 16.20
INSTRUM spect
PROBHD 5 mm CPTCI 1H-
PULPROG hmbcgplpndqf
TD 4096
SOLVENT Acetone
NS 8
DS 16
SWH 6602.113 Hz
FIDRES 1.611844 Hz
AQ 0.3102037 sec
RG 203
DW 75.733 usec
DE 6.50 usec
TE 303.2 K
CNST2 145.0000000
CNST13 10.0000000
D0 0.00000300 sec
D1 1.50000000 sec
D2 0.00344828 sec
D6 0.05000000 sec
D16 0.00020000 sec
IN0 0.00001505 sec

======== CHANNEL f1 ========
NUC1 1H
P1 8.00 usec
P2 16.00 usec
PL1 4.00 dB
PL1W 5.26999998 W
SFO1 600.1333007 MHz

======== CHANNEL f2 ========
NUC2 13C
P3 12.00 usec
PL2 0.20 dB
PL2W 84.43891907 W
SFO2 150.9201628 MHz

====== GRADIENT CHANNEL =====
GPNAM[1] SINE.100
GPNAM[2] SINE.100
GPNAM[3] SINE.100
GPZ1 50.00 %
GPZ2 30.00 %
GPZ3 40.10 %
P16 1000.00 usec

F1 - Acquisition parameters
TD 256
SFO1 150.9202 MHz
FIDRES 259.394043 Hz
SW 220.000 ppm
FnMODE QF

F2 - Processing parameters
SI 2048
SF 600.1300189 MHz
WDW SINE
SSB 2
LB 0 Hz
GB 0
PC 1.40

F1 - Processing parameters
SI 1024
MC2 QF
SF 150.9028252 MHz
WDW SINE
SSB 2
LB 0 Hz
GB 0

CPM-3
Polygonumins A, H-H Cosy (Acetone)

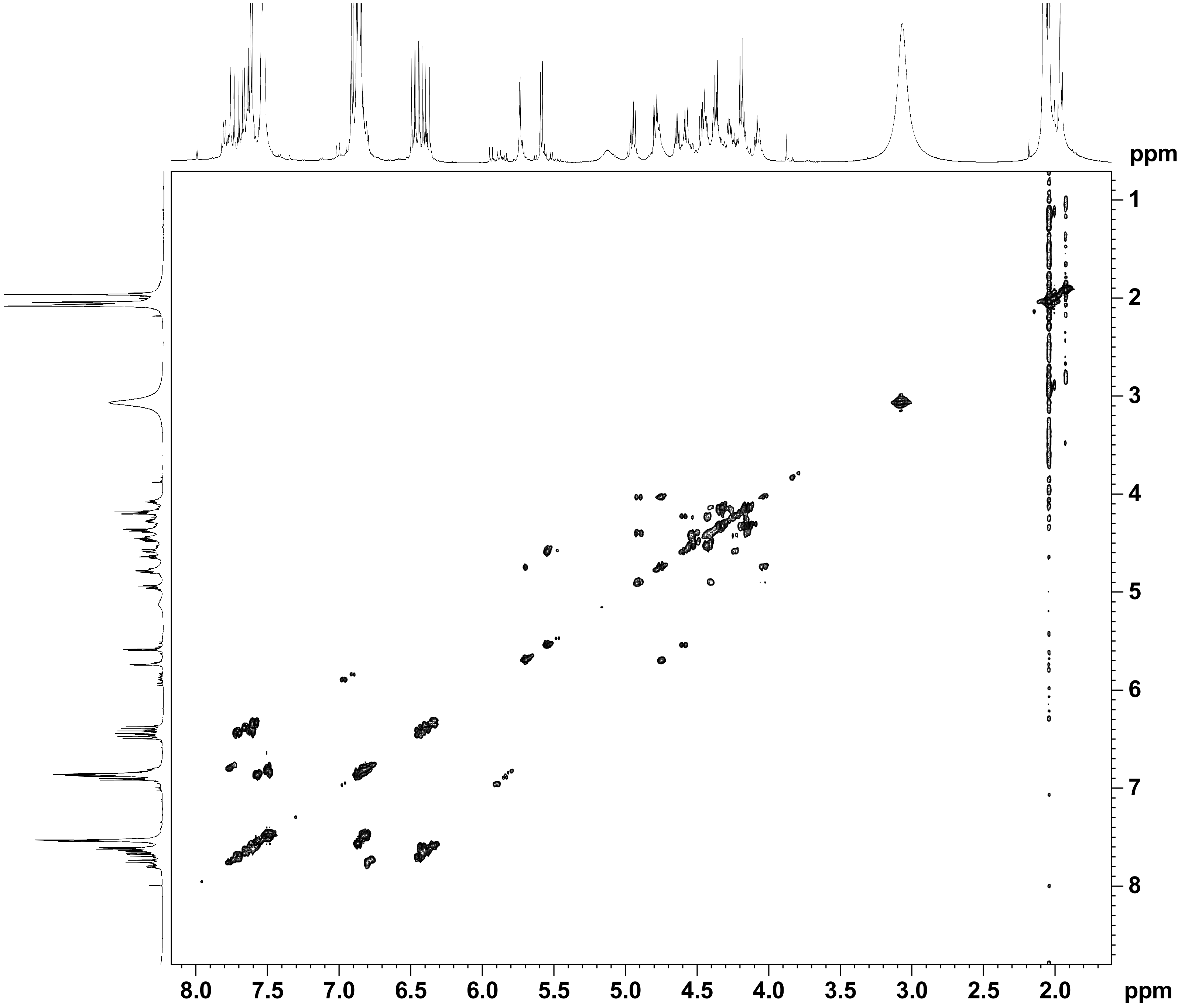

Current Data Parameters
NAME CPM-3
EXPNO 3
PROCNO 1

F2 - Acquisition Parameters
Date_ 20101008
Time 15.51
INSTRUM spect
PROBHD 5 mm CPTCI 1H-
PULPROG cosygpqf
TD 2048
SOLVENT Acetone
NS 4
DS 8
SWH 6602.113 Hz
FIDRES 3.223688 Hz
AQ 0.1551019 sec
RG 71.8
DW 75.733 usec
DE 6.50 usec
TE 303.2 K
D0 0.00000300 sec
D1 1.48689198 sec
D13 0.00000400 sec
D16 0.00020000 sec
IN0 0.00015150 sec

======== CHANNEL f1 ========
NUC1 1H
P0 8.00 usec
P1 8.00 usec
PL1 4.00 dB
PL1W 5.26999998 W
SFO1 600.1333007 MHz

====== GRADIENT CHANNEL =====
GPNAM[1] SINE.100
GPZ1 10.00 %
P16 1000.00 usec

F1 - Acquisition parameters
TD 256
SFO1 600.1333 MHz
FIDRES 51.573956 Hz
SW 11.000 ppm
FnMODE QF

F2 - Processing parameters
SI 1024
SF 600.1300431 MHz
WDW SINE
SSB 0
LB 0 Hz
GB 0
PC 1.40

F1 - Processing parameters
SI 1024
MC2 QF
SF 600.1300461 MHz
WDW SINE
SSB 0
LB 0 Hz
GB 0

CPM-3
Polygonmins A, C-H HSQC (Acetone)

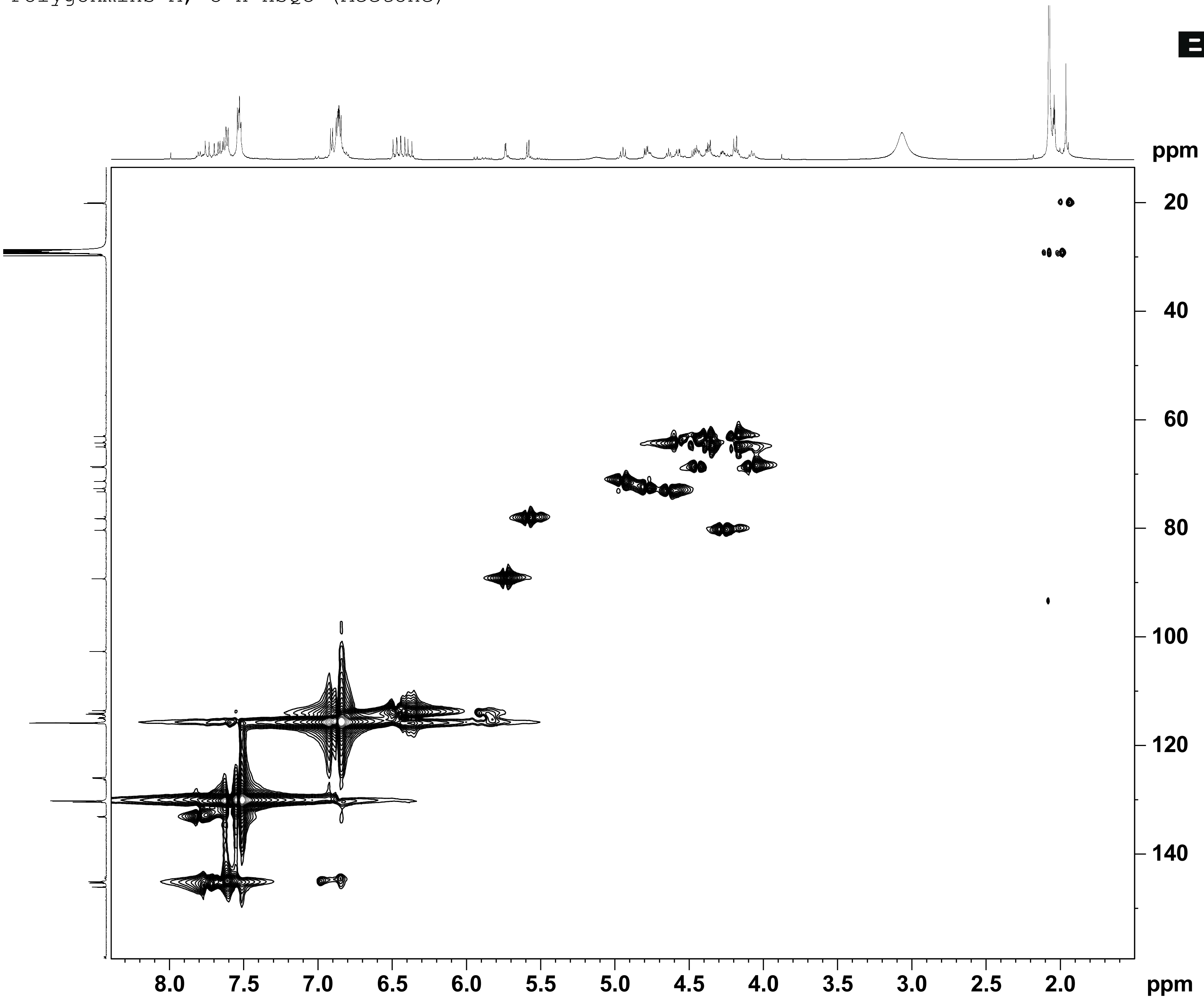


```
Current Data Parameters
NAME              CPM-3
EXPNO                 4
PROCNO                1

F2 - Acquisition Parameters
Date_          20101008
Time              17.24
INSTRUM           spect
PROBHD   5 mm CPTCI 1H-
PULPROG      hsqcedetgp
TD                 1024
SOLVENT         Acetone
NS                    8
DS                   16
SWH            6602.113 Hz
FIDRES         6.447376 Hz
AQ            0.0775509 sec
RG                  203
DW               75.733 usec
DE                 6.50 usec
TE                303.2 K
CNST2       145.0000000
D0           0.00000300 sec
D1           1.50000000 sec
D4           0.00172414 sec
D11          0.03000000 sec
D13          0.00000400 sec
D16          0.00020000 sec
D21          0.00345000 sec
IN0          0.00001505 sec
ZGOPTNS

======== CHANNEL f1 ========
NUC1                 1H
P1                 8.00 usec
P2                16.00 usec
P28                   0 usec
PL1                4.00 dB
PL1W         5.26999998 W
SFO1        600.1333007 MHz

======== CHANNEL f2 ========
CPDPRG[2           garp
NUC2                13C
P3                12.00 usec
P4                24.00 usec
PCPD2             55.00 usec
PL2                0.20 dB
PL12              13.42 dB
PL2W        84.43891907 W
PL12W        4.02293158 W
SFO2        150.9201628 MHz

====== GRADIENT CHANNEL =====
GPNAM[1]       SINE.100
GPNAM[2]       SINE.100
GPZ1              80.00 %
GPZ2              20.10 %
P16             1000.00 usec

F1 - Acquisition parameters
TD                  256
SFO1           150.9202 MHz
FIDRES       259.394043 Hz
SW              220.000 ppm
FnMODE     Echo-Antiecho

F2 - Processing parameters
SI                 1024
SF          600.1300156 MHz
WDW               QSINE
SSB                   2
LB                    0 Hz
GB                    0
PC                 1.40

F1 - Processing parameters
SI                 1024
MC2        echo-antiecho
SF          150.9028426 MHz
WDW               QSINE
SSB                   2
LB                    0 Hz
GB                    0
```
