# Supplementary material for: Bioassay Analysis and Molecular Docking Study Revealed the Potential Medicinal Activities of Active Compounds Polygonumins B, C and D from Polygonum minus (Persicaria minor)
Source: Plants (Basel). 2022 Dec 22;12(1):59. doi: 10.3390/plants12010059 (PMC9823858; doi:10.3390/plants12010059)
Supplement: Supplementary file 1 [file plants-12-00059-s001.zip › Figure S2 1H, 13C, HMBC, COSY and HSQC of Polygonumins B.pdf]

Polygonumins B in acetone
proton

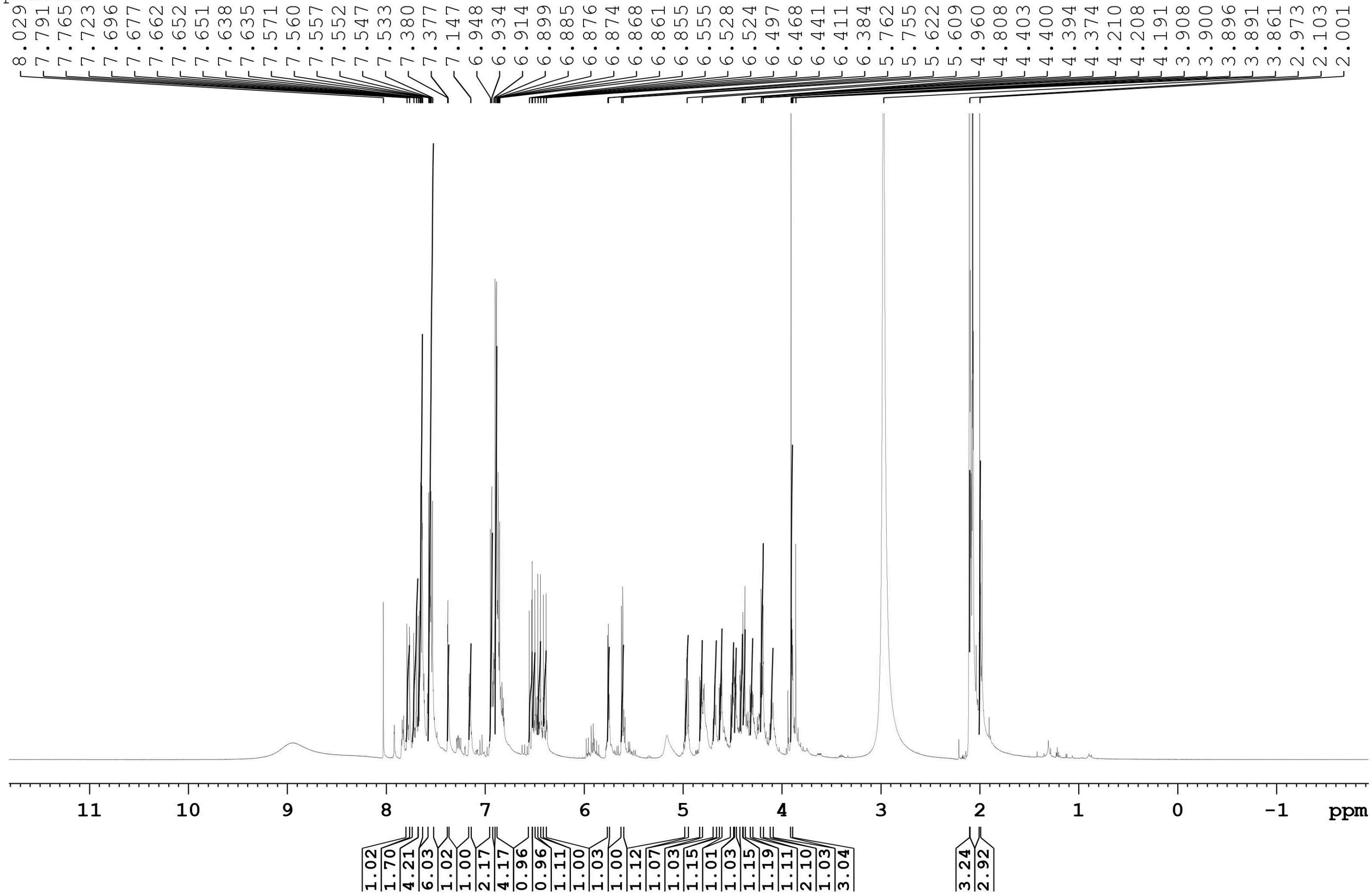

Polygonumins B in acetone
C13

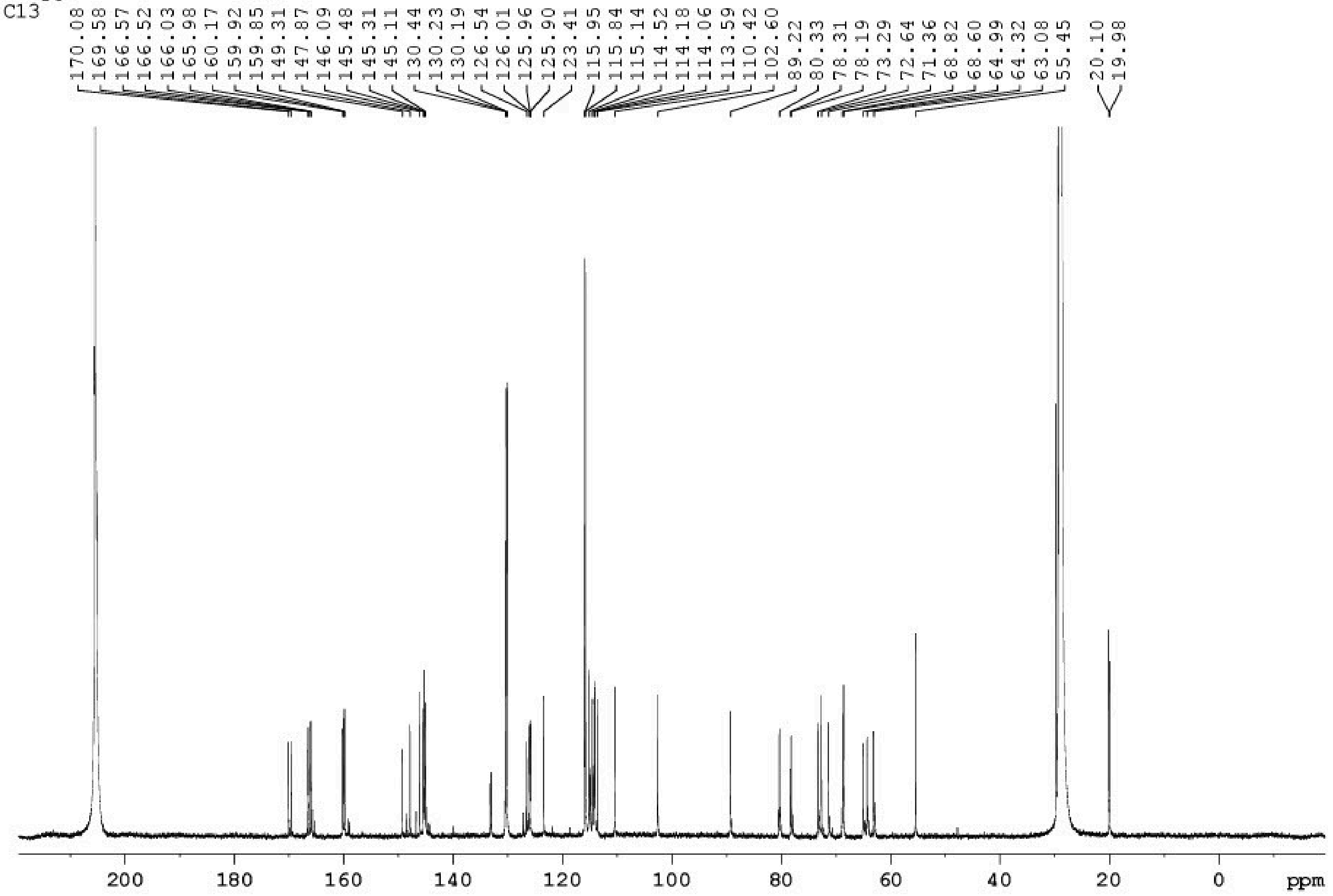

HMBC of polygonumins B

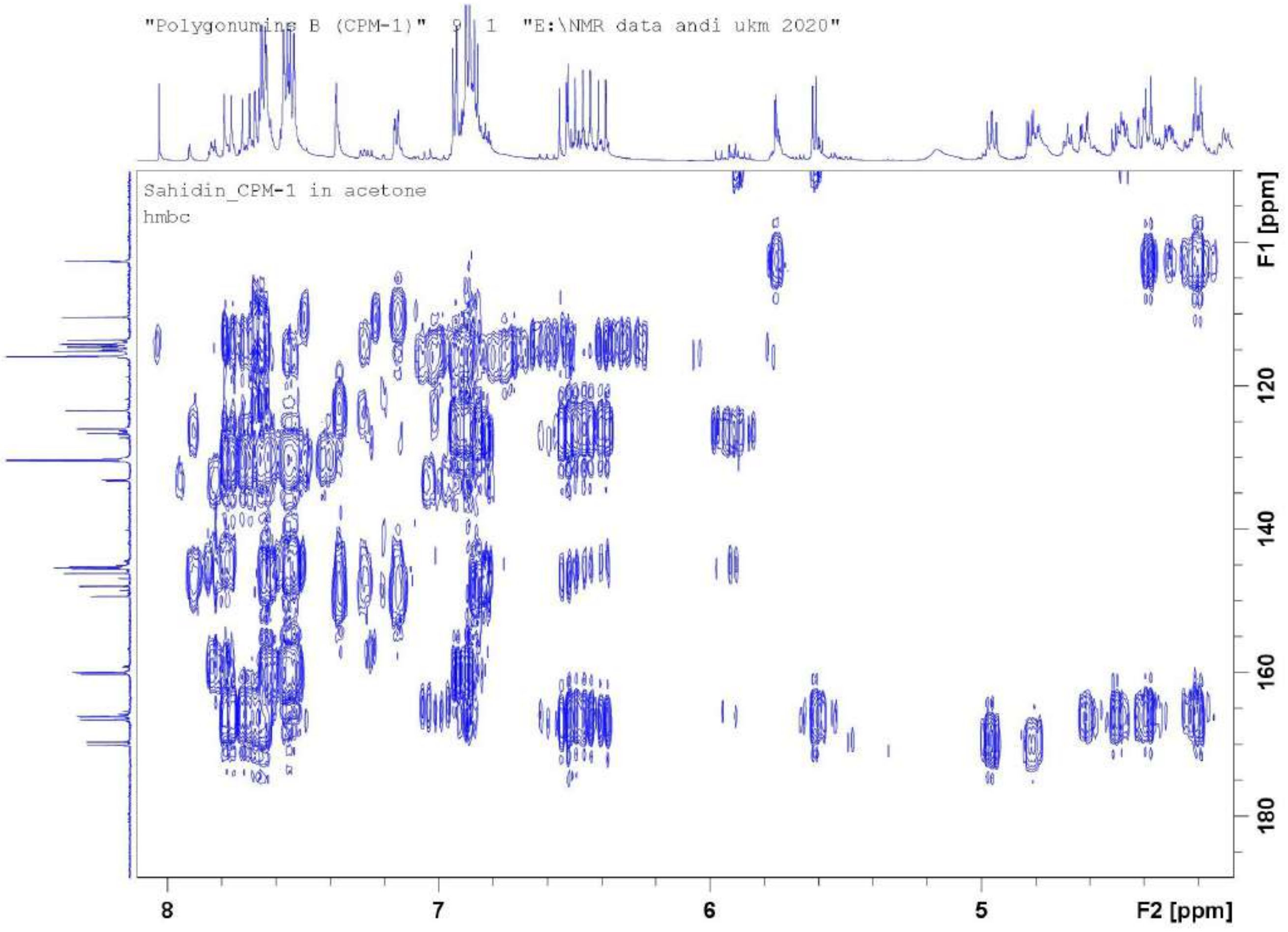

Sahidin_CPM-1 in acetone
cosy of polygonumins B in acetone

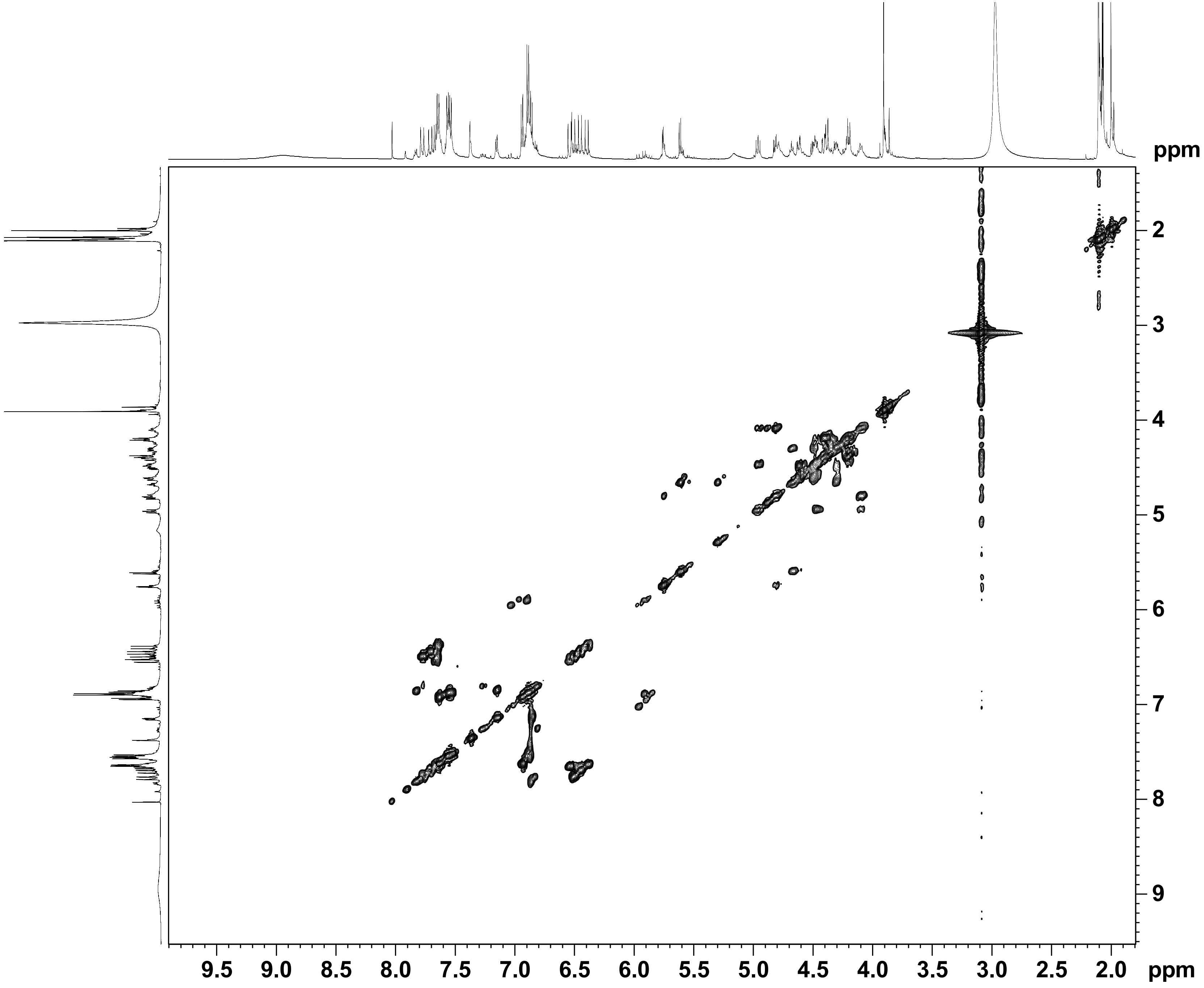


```
Current Data Parameters
NAME                CPM-1
EXPNO                   2
PROCNO                  1

F2 - Acquisition Parameters
Date_            20101026
Time                21.38
INSTRUM             spect
PROBHD   5 mm CPTCI 1H-
PULPROG          cosygpqf
TD                   2048
SOLVENT           Acetone
NS                     16
DS                     16
SWH              8012.820 Hz
FIDRES           3.912510 Hz
AQ              0.1277952 sec
RG                   90.5
DW                 62.400 usec
DE                   6.50 usec
TE                  302.9 K
D0             0.00000300 sec
D1             1.48689198 sec
D13            0.00000400 sec
D16            0.00020000 sec
IN0            0.00012480 sec

======== CHANNEL f1 ========
NUC1                   1H
P0                   8.00 usec
P1                   8.00 usec
PL1                  4.00 dB
PL1W           5.26999998 W
SFO1          600.1336081 MHz

====== GRADIENT CHANNEL =====
GPNAM[1]         SINE.100
GPZ1                10.00 %
P16               1000.00 usec

F1 - Acquisition parameters
TD                    256
SFO1             600.1336 MHz
FIDRES          62.593380 Hz
SW                 13.350 ppm
FnMODE                 QF

F2 - Processing parameters
SI                   1024
SF            600.1300000 MHz
WDW                  SINE
SSB                     0
LB                      0 Hz
GB                      0
PC                   1.40

F1 - Processing parameters
SI                   1024
MC2                    QF
SF            600.1300000 MHz
WDW                  SINE
SSB                     0
LB                      0 Hz
GB                      0
```

CPM-1

HSQC of Polygonumins-B in acetone

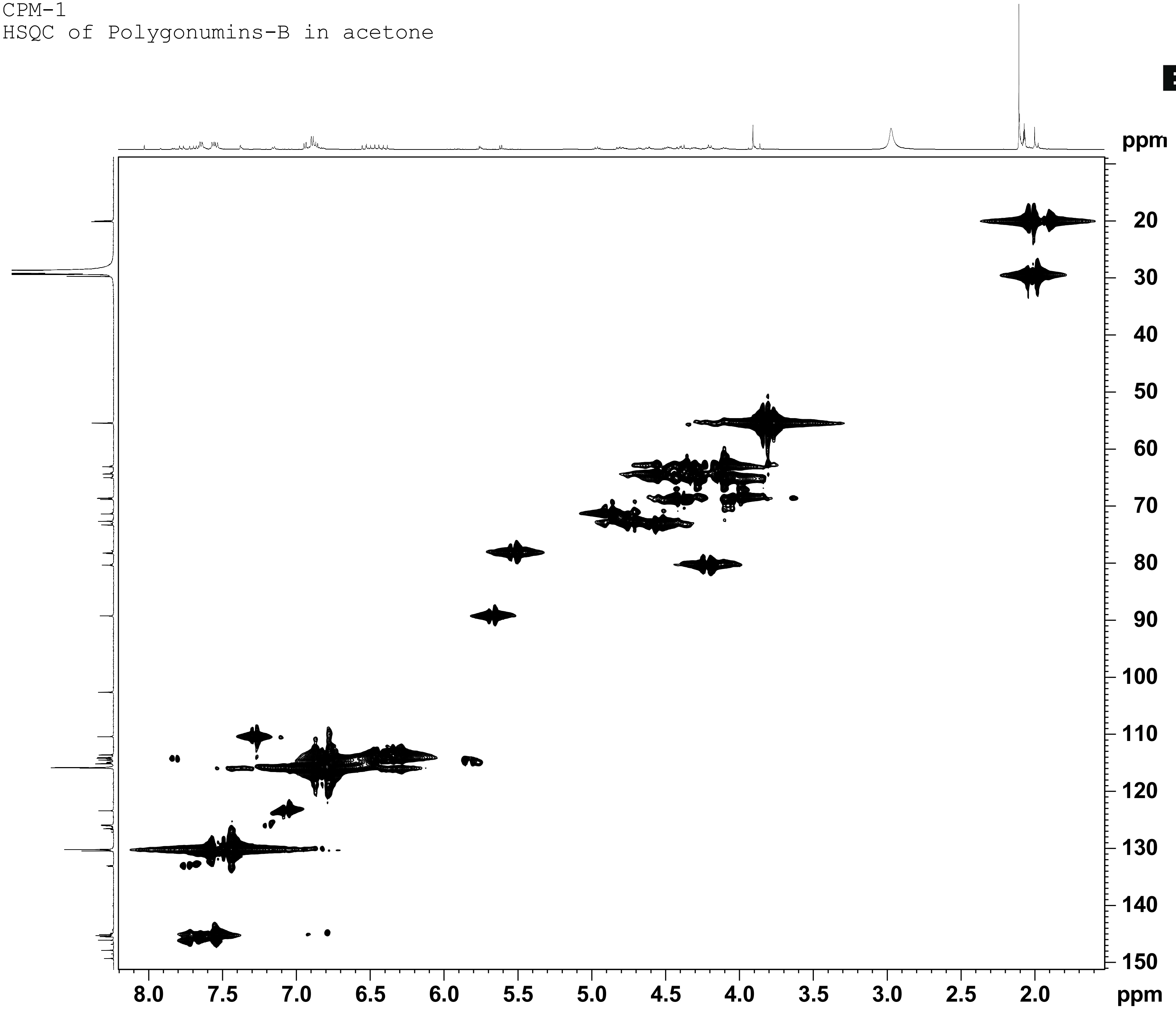


```
Current Data Parameters
NAME                  CPM-1
EXPNO                     8
PROCNO                    1

F2 - Acquisition Parameters
Date_              20101022
Time                  18.23
INSTRUM               spect
PROBHD   5 mm CPTCI 1H-
PULPROG         hsqcedetgp
TD                     1024
SOLVENT             Acetone
NS                        8
DS                       16
SWH                6602.113 Hz
FIDRES             6.447376 Hz
AQ                0.0775509 sec
RG                      203
DW                   75.733 usec
DE                     6.50 usec
TE                    305.6 K
CNST2           145.0000000
D0               0.00000300 sec
D1               1.50000000 sec
D4               0.00172414 sec
D11              0.03000000 sec
D13              0.00000400 sec
D16              0.00020000 sec
D21              0.00345000 sec
IN0              0.00002000 sec
ZGOPTNS

======== CHANNEL f1 ========
NUC1                     1H
P1                     7.80 usec
P2                    15.60 usec
P28                       0 usec
PL1                    4.00 dB
PL1W             5.26999998 W
SFO1           600.1330006 MHz

======== CHANNEL f2 ========
CPDPRG[2                garp
NUC2                     13C
P3                    12.00 usec
P4                    24.00 usec
PCPD2                 55.00 usec
PL2                    0.20 dB
PL12                  13.42 dB
PL2W            84.43891907 W
PL12W            4.02293158 W
SFO2           150.9140636 MHz

====== GRADIENT CHANNEL =====
GPNAM[1]           SINE.100
GPNAM[2]           SINE.100
GPZ1                  80.00 %
GPZ2                  20.10 %
P16                 1000.00 usec

F1 - Acquisition parameters
TD                      256
SFO1               150.9141 MHz
FIDRES           195.291351 Hz
SW                  165.639 ppm
FnMODE         Echo-Antiecho

F2 - Processing parameters
SI                     1024
SF              600.1300469 MHz
WDW                   QSINE
SSB                       2
LB                        0 Hz
GB                        0
PC                     1.40

F1 - Processing parameters
SI                     1024
MC2            echo-antiecho
SF              150.9028090 MHz
WDW                   QSINE
SSB                       2
LB                        0 Hz
GB                        0
```
