# Supplementary material for: Bioassay Analysis and Molecular Docking Study Revealed the Potential Medicinal Activities of Active Compounds Polygonumins B, C and D from Polygonum minus (Persicaria minor)
Source: Plants (Basel). 2022 Dec 22;12(1):59. doi: 10.3390/plants12010059 (PMC9823858; doi:10.3390/plants12010059)
Supplement: Supplementary file 1 [file plants-12-00059-s001.zip › Figure S3 1H,13C, HMBC, COSY and HSQC of Polygonumins C.pdf]

Polygonumins C in acetone
proton

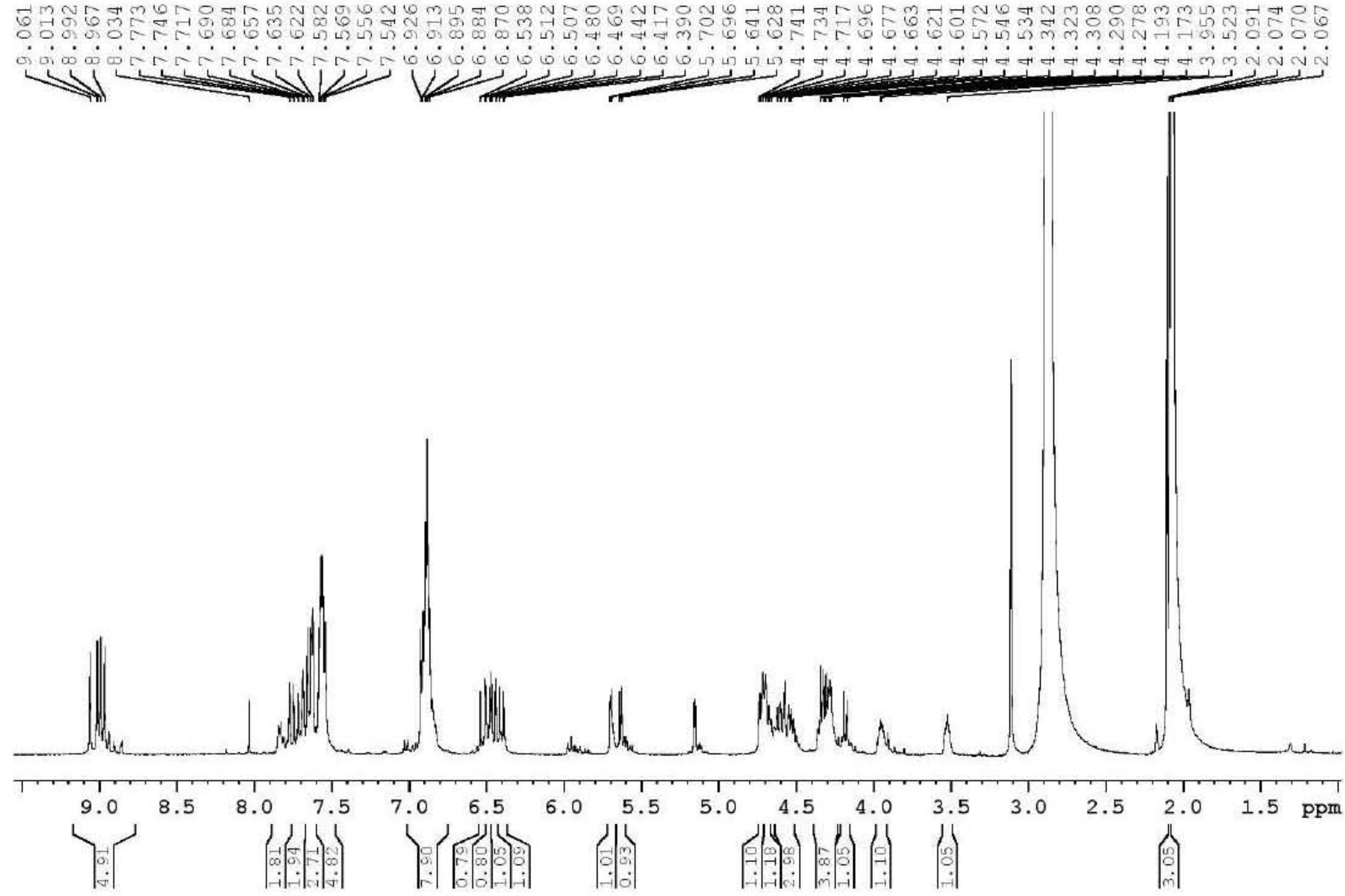

Polygonumins C in acetone

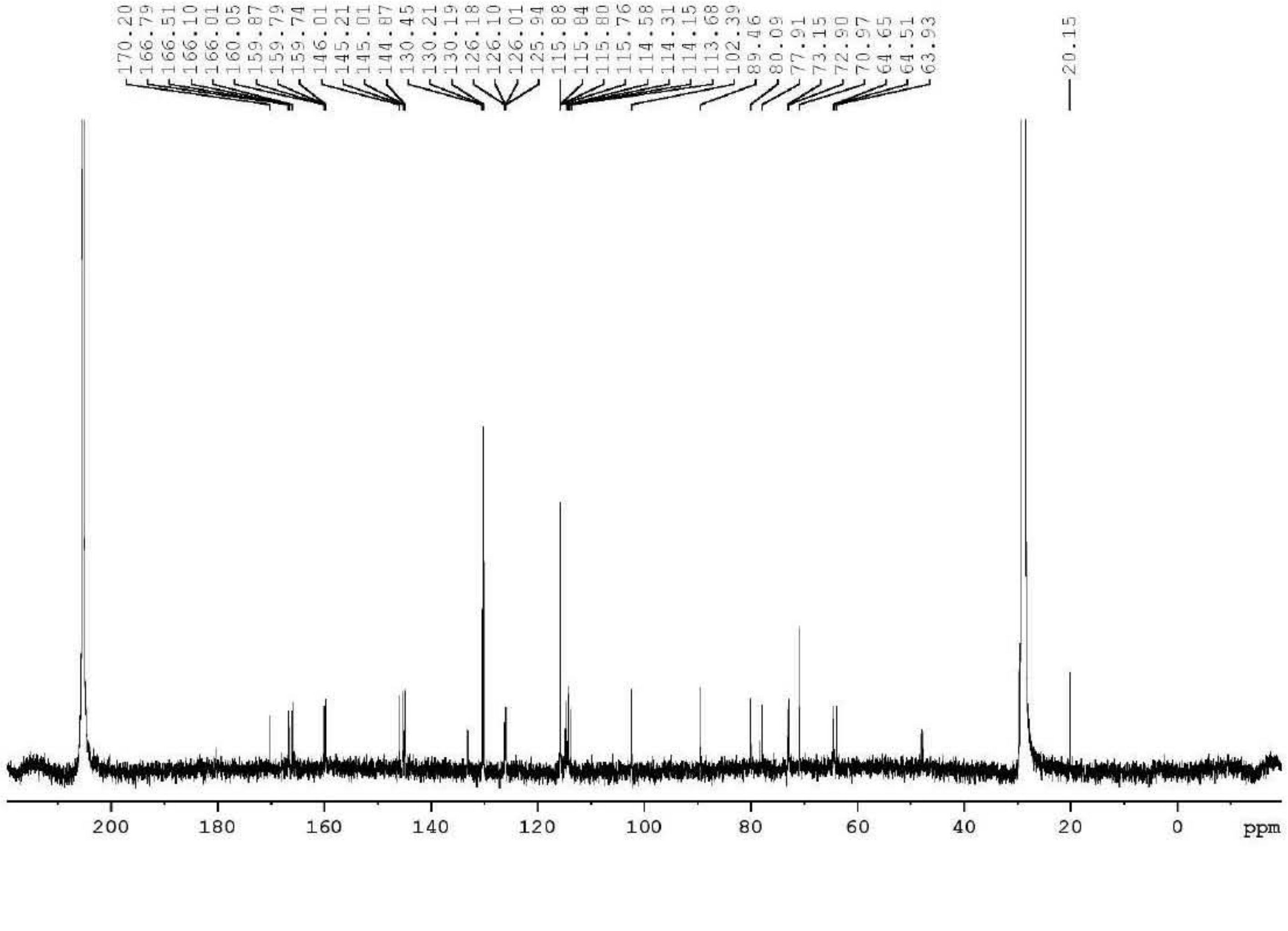

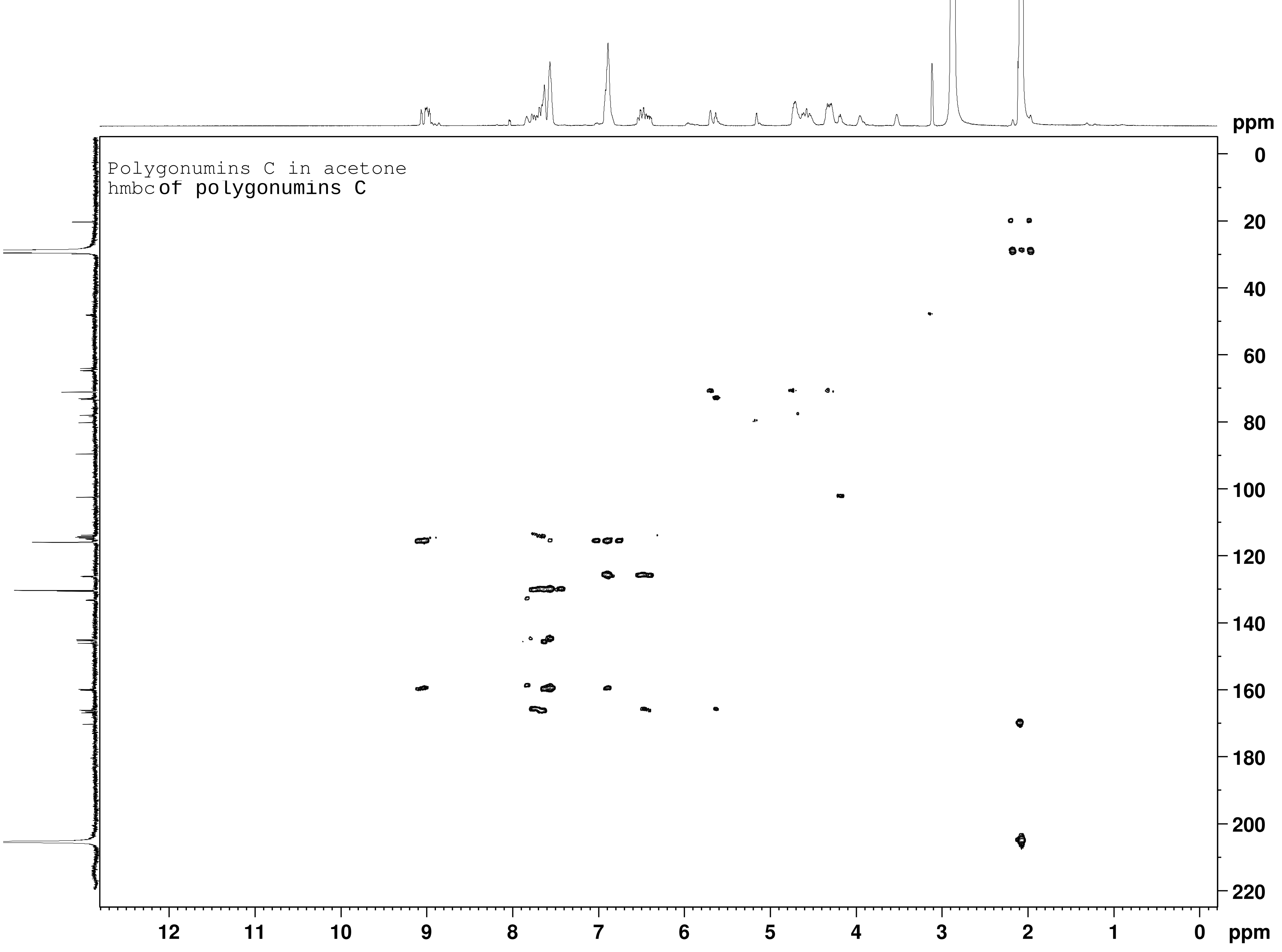

Polygonumins C in acetone
hmbc of polygonumins C
ppm
0
20
40
60
80
100
120
140
160
180
200
220
12
11
10
9
8
7
6
5
4
3
2
1
0
ppm

CPM-5
Cosy of Polygonumins-C in acetone

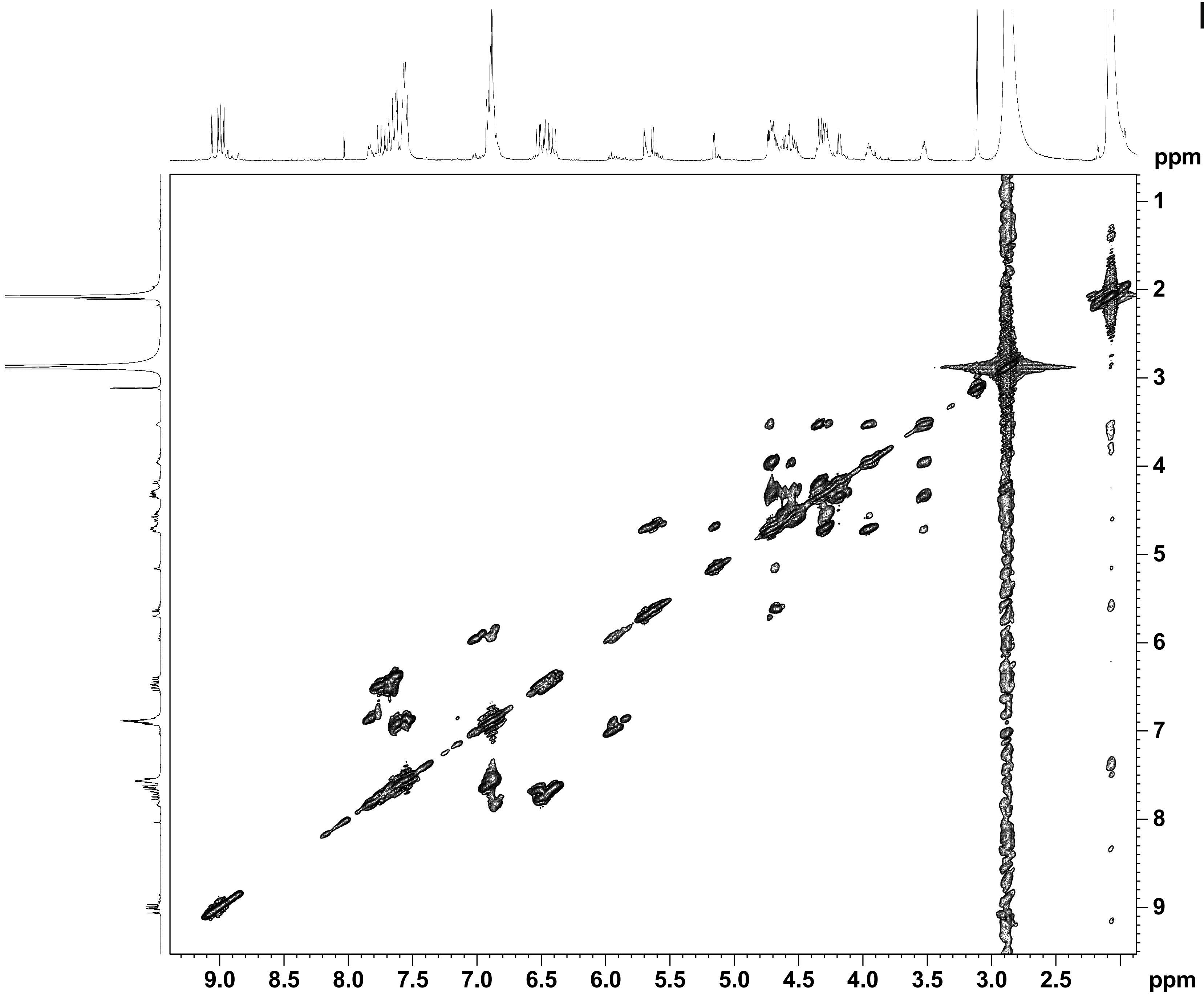

Current Data Parameters
NAME CPM-5
EXPNO 4
PROCNO 1

F2 - Acquisition Parameters
Date_ 20101027
Time 23.28
INSTRUM spect
PROBHD 5 mm CPTCI 1H-
PULPROG cosygpqf
TD 2048
SOLVENT Acetone
NS 16
DS 16
SWH 8012.820 Hz
FIDRES 3.912510 Hz
AQ 0.1277952 sec
RG 203
DW 62.400 usec
DE 6.50 usec
TE 306.2 K
D0 0.00000300 sec
D1 1.48689198 sec
D13 0.00000400 sec
D16 0.00020000 sec
IN0 0.00012480 sec

======== CHANNEL f1 ========
NUC1 1H
P0 8.00 usec
P1 8.00 usec
PL1 4.00 dB
PL1W 5.26999998 W
SFO1 600.1336081 MHz

====== GRADIENT CHANNEL =====
GPNAM[1] SINE.100
GPZ1 10.00 %
P16 1000.00 usec

F1 - Acquisition parameters
TD 256
SFO1 600.1336 MHz
FIDRES 62.593380 Hz
SW 13.350 ppm
FnMODE QF

F2 - Processing parameters
SI 1024
SF 600.1300000 MHz
WDW SINE
SSB 0
LB 0 Hz
GB 0
PC 1.40

F1 - Processing parameters
SI 1024
MC2 QF
SF 600.1300000 MHz
WDW SINE
SSB 0
LB 0 Hz
GB 0

CPM-5

HSQC of Polygonumins-C in acetone

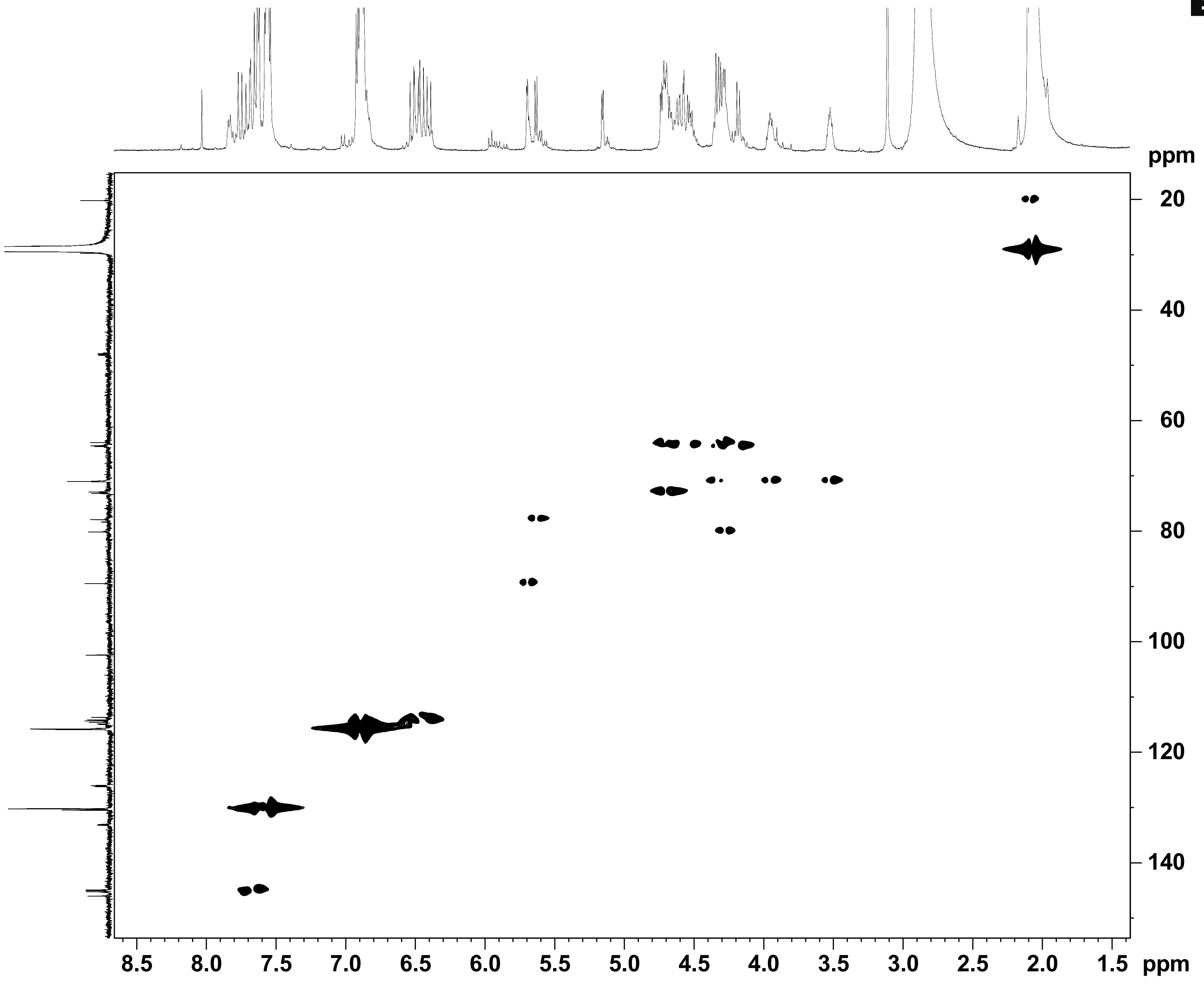


```
Current Data Parameters
NAME                 CPM-5
EXPNO                    5
PROCNO                   1

F2 - Acquisition Parameters
Date_             20101027
Time                  1.26
INSTRUM              spect
PROBHD     5 mm CPTCI 1H-
PULPROG         hsqcedetgp
TD                    1024
SOLVENT            Acetone
NS                      16
DS                      16
SWH               8012.820 Hz
FIDRES            7.825020 Hz
AQ               0.0638976 sec
RG                     203
DW                  62.400 usec
DE                    6.50 usec
TE                   302.9 K
CNST2          145.0000000
D0              0.00000300 sec
D1              1.50000000 sec
D4              0.00172414 sec
D11             0.03000000 sec
D13             0.00000400 sec
D16             0.00020000 sec
D21             0.00345000 sec
IN0             0.00001885 sec
ZGOPTNS

======== CHANNEL f1 ========
NUC1                    1H
P1                    8.00 usec
P2                   16.00 usec
P28                      0 usec
PL1                   4.00 dB
PL1W            5.26999998 W
SFO1           600.1336081 MHz

======== CHANNEL f2 ========
CPDPRG[2              garp
NUC2                   13C
P3                   12.00 usec
P4                   24.00 usec
PCPD2                55.00 usec
PL2                   0.20 dB
PL12                 13.42 dB
PL2W           84.43891907 W
PL12W           4.02293158 W
SFO2           150.9156357 MHz

====== GRADIENT CHANNEL =====
GPNAM[1]         SINE.100
GPNAM[2]         SINE.100
GPZ1                 80.00 %
GPZ2                 20.10 %
P16                1000.00 usec

F1 - Acquisition parameters
TD                     256
SFO1              150.9156 MHz
FIDRES          207.083725 Hz
SW                 175.639 ppm
FnMODE      Echo-Antiecho

F2 - Processing parameters
SI                    1024
SF             600.1299977 MHz
WDW                  QSINE
SSB                      2
LB                       0 Hz
GB                       0
PC                    1.40

F1 - Processing parameters
SI                    1024
MC2         echo-antiecho
SF             150.9028398 MHz
WDW                  QSINE
SSB                      2
LB                       0 Hz
GB                       0
```
